# Graphene Nanomaterials: Synthesis, Biocompatibility, and Cytotoxicity

**DOI:** 10.3390/ijms19113564

**Published:** 2018-11-12

**Authors:** Chengzhu Liao, Yuchao Li, Sie Chin Tjong

**Affiliations:** 1 Department of Materials Science and Engineering, Southern University of Science and Technology, Shenzhen 518055, China; 2 Department of Materials Science and Engineering, Liaocheng University, Liaocheng 252000, China; liyuchao@lcu.edu.cn; 3 Department of Physics, City University of Hong Kong, Tat Chee Avenue, Kowloon, Hong Kong, China

**Keywords:** graphene, synthesis, cell culture, biocompatibility, toxicity, impurities, apoptosis, in vitro, in vivo, oxidative stress

## Abstract

Graphene, graphene oxide, and reduced graphene oxide have been widely considered as promising candidates for industrial and biomedical applications due to their exceptionally high mechanical stiffness and strength, excellent electrical conductivity, high optical transparency, and good biocompatibility. In this article, we reviewed several techniques that are available for the synthesis of graphene-based nanomaterials, and discussed the biocompatibility and toxicity of such nanomaterials upon exposure to mammalian cells under in vitro and in vivo conditions. Various synthesis strategies have been developed for their fabrication, generating graphene nanomaterials with different chemical and physical properties. As such, their interactions with cells and organs are altered accordingly. Conflicting results relating biocompatibility and cytotoxicity induced by graphene nanomaterials have been reported in the literature. In particular, graphene nanomaterials that are used for in vitro cell culture and in vivo animal models may contain toxic chemical residuals, thereby interfering graphene-cell interactions and complicating interpretation of experimental results. Synthesized techniques, such as liquid phase exfoliation and wet chemical oxidation, often required toxic organic solvents, surfactants, strong acids, and oxidants for exfoliating graphite flakes. Those organic molecules and inorganic impurities that are retained in final graphene products can interact with biological cells and tissues, inducing toxicity or causing cell death eventually. The residual contaminants can cause a higher risk of graphene-induced toxicity in biological cells. This adverse effect may be partly responsible for the discrepancies between various studies in the literature.

## 1. Introduction

Nanotechnology is a multidisciplinary field of research spanning several diverse disciplines, such as biology, chemistry, engineering, materials, medical science, and physics. In particular, nanotechnology involves the creation and development of novel materials and devices through the manipulation of properties and functions of matter at the nanometer scale. At this length scale, the chemical, mechanical, physical, and biological behaviors of materials differ substantially from their bulk matter counterparts. Nanotechnology holds great promise for the development and synthesis of nanomaterials with unique chemical, biological, mechanical, and physical properties. By introducing nanotechnology in medical sector, scientists are capable of developing new materials and strategies for accurate detection and prevention of malignant diseases, novel medical implants and artificial prosthesis with good biocompatibility [1,2,3].

Carbon-based materials such as carbon nanotubes and graphene are widely known to possess exceptionally high Young’s modulus and mechanical strength, high light transmittance, and excellent electrical conductivity [4,5,6,7,8,9]. Such attractive properties have resulted in widespread interest in the use of these materials for making electronic devices, composite materials, drug deliveries, medical implants, etc. [10,11,12,13,14,15,16,17,18]. For instance, one-dimensional (1D) carbon nanotubes (CNTs) are promising candidates to deliver drugs in biological cells due to their hollow structure. CNTs with a needle-like feature can penetrate plasma membrane easily, thereby transporting therapeutic molecules effectively. However, cell penetrating nanotubes can also be harmful to biological cells because they induce appreciable toxicity and apoptosis [19,20,21]. Electron microscopic observations have revealed the presence of nanotubes in the cytoplasm, thereby inducing oxidative stress, reducing metabolic activity, and causing eventual cell death [22,23]. In this respect, the application of carbon nanotubes in clinical field is hampered by their biodistribution, size, and shape [19,20,21].

**Figure 1:**
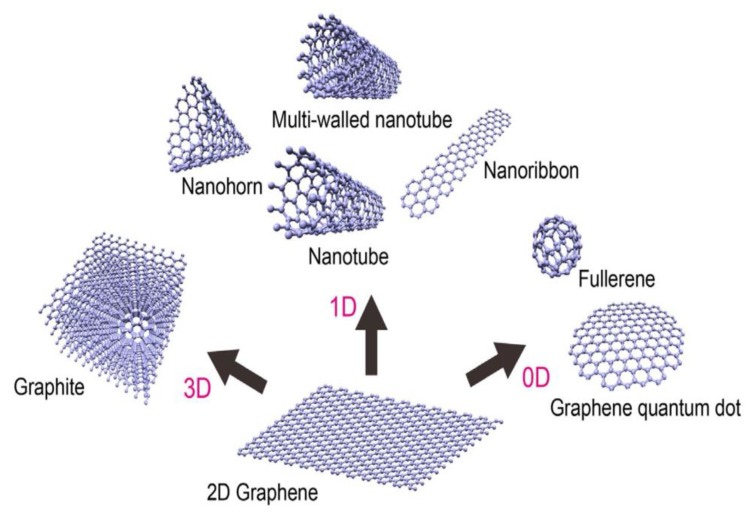
Carbonaceous materials of different dimensionalities. Reproduced from [24] with permission of Taylor & Francis.

Graphene is a two-dimensional monolayer of sp^2^ hybridized carbon atoms bonded covalently in a hexagonal lattice. It is a basic building block for carbon materials of all other dimensionalities, including 0D Bucky ball and graphene quantum dot (GQD), 1D carbon nanotube, and 3D graphite (Figure 1) [24]. Graphene was firstly isolated from graphite by Geim and Novoselov through mechanical cleavage using a scotch-tape to attach flakes of graphene layers [25]. Mechanically exfoliated graphene sheet is pure and defect free, but its yield is very small. Its application is limited to research only for studying electrical, mechanical, and optical properties of pure graphene. Therefore, numerous studies have been conducted by the researchers to synthesize graphene at a large scale, including its derivatives such as graphene oxide (GO), reduced graphene oxide (rGO), and thermally reduced graphene oxide (TRG) [14,26,27,28,29,30,31,32,33,34]. The remarkable electrical, mechanical and optical properties of graphene sheets, and GQDs render them attractive materials for use in electronic, optoelectronic, and aerospace industries [35,36,37].

**Figure 2:**
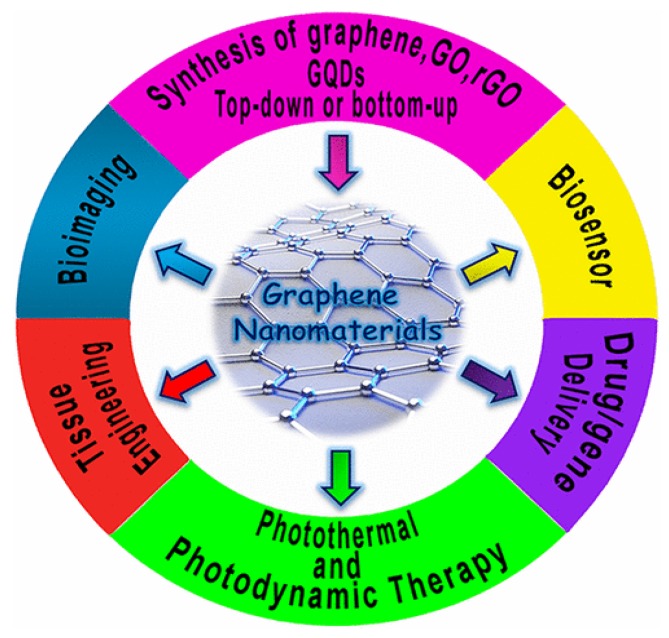
Applications of graphene nanomaterials in biomedical engineering. Reproduced from [40] with permission of the American Chemical Society.

Graphene and its derivatives as well as GQDs have also emerged rapidly as promising materials for use in the biomedical sector, such as biosensors, tissue engineering, bioimaging, drug delivery, and photothermal therapy (Figure 2) [18,38,39,40,41,42]. GQDs generally exhibit photoluminescence due to a quantum confinement effect. So, GQDs find applications in the biomedical field for imaging and sensor purposes. For instance, plant-leaf-derived GQDs with attractive photoluminescent properties are particularly suitable for bioimaging, including zebrafish and human cancer cell lines. Zebrafish can grow healthily in the presence of GQDs (< 2 mg/mL) [2,42]. The main concern in using graphene-based materials for biomedical applications is their biocompatibility. From established works in literature, the biocompatibility of graphene and its derivatives are often contradictory. The effects of graphene nanomaterials on cell viability depend on various factors and experimental conditions [43,44,45]. Accordingly, it is necessary to investigate the biocompatibility and toxicity of graphene and its derivatives through in vitro cell cultures and in vivo animal models. Chang et al. found that GO does not enter A549 cells (human alveolar basal epithelial cells) and it shows no obvious cytotoxicity. However, GO tends to induce a dose-dependent oxidative stress in cells, causing a small reduction of cell viability at high concentration. These effects are dose and size related [46]. Similarly, Wang et al. reported that GO exhibits dose-dependent toxicity to human fibroblast cells and mice [47]. Their results revealed that GO with doses smaller than 20 μg/mL exhibit no toxicity to human fibroblast cells. At doses higher than 50 μg/mL, obvious cytotoxicity, such as reduction of cell adhesion and cell apoptosis, is observed. For in vivo mice tests, low dose (0.1 mg) and middle dose (0.25 mg) GO shows no obvious toxicity, but a high dose (0.4 mg) causes chronic toxicity, leading to mice death and lung granuloma formation. Yang et al. investigated in vivo biodistribution of graphene functionalized with polyethylene glycol (PEG) in mice. They indicated that PEGylated graphene does not induce appreciable toxicity under a dose of 20 mg/kg for three months [48]. This review summarizes the synthesis of graphene and its derivatives, and the effects of interactions between graphene-based nanomaterials with mammalian cells under in vitro and in vivo conditions. The interactions of graphene nanomaterials with biological cells is crucial for biosafety evaluation to assure their safe use in biomedical sectors.

## 2. Synthesis of Graphene-Based Nanomaterials

**Figure 3:**
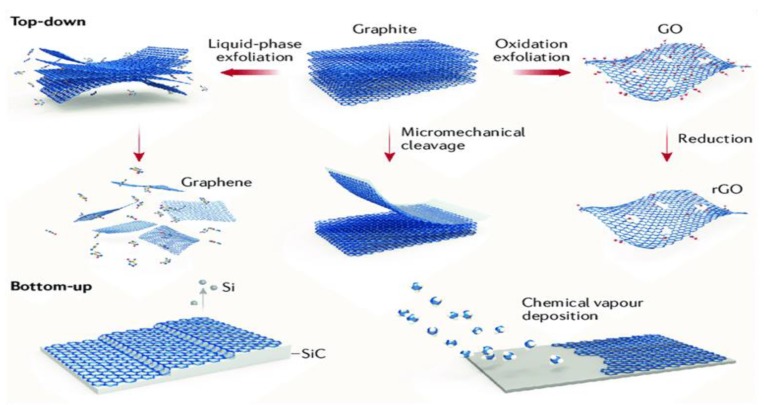
Several methods used for fabricating graphene. Reproduced from [49] with permission of Springer Nature.

Graphene generally can be synthesized from both top-down and bottom-up routes. The top-down route includes micromechanical cleavage of graphite, liquid-phase exfoliation, and chemical exfoliation of graphite to produce GO, followed by chemical or thermal treatments to obtain rGO or TRG, respectively. The bottom-up fabrication route includes chemical vapor deposition and epitaxial growth on the SiC substrate (Figure 3) [49].

### 2.1. Epitaxial Graphene on SiC Wafers

Graphene films can be grown on SiC wafers through surface sublimation of Si atoms from the wafers in ultrahigh vacuum (UHV) at high temperatures (typically above 1000 °C). As a result, excess carbon domains are left behind on the wafer surface and are then reconstructed to produce graphene [50,51]. However, the high cost and small size of SiC wafers and the need of UHV system at high temperatures impede the adoption of this process for large-scale production of graphene in industries.

### 2.2. Chemical Vapor Deposited Graphene Films

**Figure 4:**
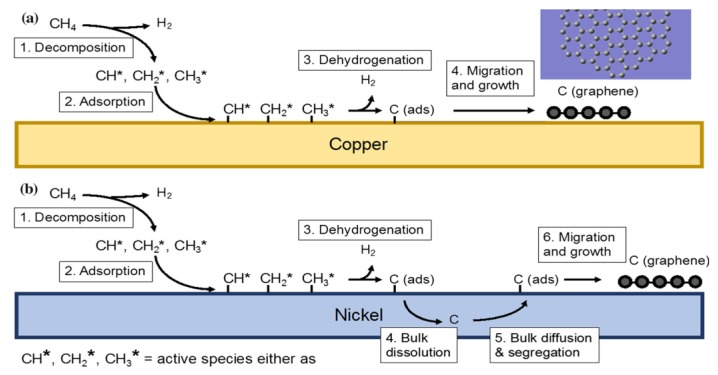
Schematic diagram showing deposition of graphene on (**a**) copper and (**b**) nickel substrates. Reproduced from [54] with permission of the American Chemical Society.

Chemical vapor deposition (CVD) is typically employed to fabricate large-area, monolayer graphene films on transition metals (Fe, Ni, Co, Pt, Ru, etc.) by admitting hydrocarbon gas, such as methane, ethane, or propane into the reaction chamber at high temperatures [52,53]. Cu or Ni substrates are commonly used due to their low cost, and they serve as a catalyst for hydrocarbon gas decomposition (Figure 4) [54]. The thin films were then transferred onto target substrates such as SiO_2_/Si, glass or flexible polymer such as poly(ethylene terephthalate) (PET). The deposition and growth of graphene films takes place in two steps. The first step involves an initial pyrolysis of the precursor to form carbon. This is followed by the formation of graphitic structure from dissociated carbon atoms. In general, CVD growth of graphene on Cu substrate with low carbon solubility occurs through a surface adsorption mechanism. In the process, the carbon precursor decomposes and it adsorbs only on the metal surface, followed by migration and growth. In contrast, graphene on Ni takes place through carbon segregation and precipitation [52,53,54]. Carbon species decompose from hydrocarbon gas and diffuse into nickel metal surface with high carbon solubility at elevated temperatures to create a solid solution. During cooling, carbon solubility decreases, allowing for carbon atoms to diffuse out of the metal and then precipitate on the Ni surface to produce graphene. Several factors can affect the growth and quality of graphene films including the types of substrate materials, and CVD processing parameters such as precursor gas type, concentration, flow rate, and temperature [55]. The graphene films that are formed by randomly oriented graphene islands are polycrystalline having a high density of grain boundaries. Those grain boundary defects degrade electrical properties of graphene films greatly since they serve as the scattering centers for electrons, resulting in reduced mobility as expected. In this respect, it is desired to fabricate graphene films with large grains with fewer grain boundaries, or even single-crystal graphene films. Very recently, Xu et al. fabricated large-area, single-crystal graphene (5 × 50 cm^2^) on the meter-sized single-crystal Cu(111) foil through epitaxial growth of graphene islands on copper surface [56]. The as-synthesized graphene film had a mobility up to 23,000 cm^2^ V^−1^ s^−1^ at 4 K. High quality CVD-grown graphene films find attractive applications in making bendable touch panels, displays, and optoelectronic devices.

In general, direct transfer of graphene film from Cu or Ni foils to a target substrate such as SiO_2_/Si, glass or PET is needed for practical applications. Such a graphene transferring process can induce defects, such as wrinkles and cracks, which are detrimental to its electrical and optical properties. To eliminate the transfer process, direct graphene synthesis on nonmetalic materials such as hexagonal boron nitride, without a need to transfer to another substrate has been explored. However, direct graphene growth is very slow and the final size of graphene film is also limited in the absence of transition metal Cu or Ni catalyst. Accordingly, the high cost of production and tedious film transferring process are the major obstacles for CVD-graphene commercialization at present [57]. As thermal CVD requires high temperature of 800–1000 °C for growing graphene films, it is of practical interest to reduce processing temperatures or pressures, thereby permitting large-area fabrication with lower cost [28]. Reduced pressures can minimize unwanted gas-phase reactions and achieve film uniformity over the whole substrate. Ruoff and coworkers demonstrated that single crystal graphene films can be synthesized by low-pressure chemical vapor deposition (LPCVD) in copper-foil enclosures using methane as a precursor [58]. Those LPCVD films exhibited superior carrier mobility.

### 2.3. Liquid Phase Exfoliation

Liquid-phase exfoliation (LPE) is a scalable route for the mass production of graphene due to its low cost and simplicity. In the process, graphite is dispersed in a solvent to weaken the van der Waals forces between graphene interlayers in the presence or absence of surfactants. Ultrasonication or shearing can be employed to facilitate exfoliation of graphite into graphene sheets. This is followed by a purification step to yield single and multi-layered graphene sheets [30]. This strategy allows for the synthesis of exfoliated graphene sheets in the form of solvent suspension. Since surfactants, organic solvents, and strong acids are used to exfoliate and stabilize graphene flakes in the specific medium, they can cause environmental pollution problems. In addition, residual surfactants are difficult to remove from graphene sheets. Most organic solvents (e.g., *N*,*N*-dimethyl-formamide (DMF); *N*-methyl-2-pyrrolidone (NMP); dichlorobenzene (DCB)) used are highly toxic; they can induce cellular toxicity; and, should be avoided for cell manipulation purposes.

### 2.4. Chemical and Thermal Reduction of GO

Graphene oxide is a graphene derivative produced by chemical oxidation of graphite flakes in strong oxidizing agents. Modified Hummers process is widely used to produce GO by reacting graphite flakes with a mixture of sulfuric acid, sodium nitrate, and potassium permanganate under vigorous stirring or sonication [59]. The suspension is diluted with water, hydrogen peroxide is then added to achieve a higher oxidation degree, followed by rinsing with water. The drawbacks of this process are the long processing times and the generation of toxic gases (NO_2_ and N_2_O_4_). To address these issues, Tour and coworkers modified this method by substituting sodium nitrate with phosphoric acid under a mixed H_2_SO_4_/H_3_PO_4_ (9:1) ratio while increasing KMnO_4_ concentration [60]. The advantage of this method is the elimination of toxic gas formation. The disadvantages include the consumption of a large amount of KMnO_4_, and tedious sifting, filtration, centrifugation, and washing procedures. Therefore, several tactics have been developed to further modify the Hummers process, such as the employment of potassium ferrate (K_2_FeO_4_) as a strong oxidant instead of KMnO_4_ and the elimination of NaNO_3_ in the GO preparation [61].

GO is generally decorated with oxygen functional groups, such as carboxyl, hydroxyl, and epoxide [32,34]. Thus, GO sheets are amphipathic, having both hydrophilic and hydrophobic components [62]. They are easily hydrated upon exposure to water due to the presence of carboxyl groups at the edges. The oxygen functional groups also enable GO to be dispersed in several organic solvents such as acetone, DCB, NMP and DMF [63]. In addition to the above-mentioned problems, Hummer process still suffers from certain flaws including environmental issues relating to the use of strong oxidizers, and the introduction of impurities to the GO products. Furthermore, a large amount of waste acids and oxidizing agents can lead to environmental pollution on large-scale production of GOs in industries. In terms of health issue, a trace amount of Mn in GOs derived from potassium permanganate can deteriorate their physical properties and induce toxicity in mammalian cells [64]. In this respect, green synthesis of GOs has attracted considerable attention in recent years, especially in biomedical field [65]. GO is an electrical insulator, and its conductivity can be partially recovered by chemical reduction in reducing agents, such as hydrazine and sodium borohydride, to yield rGO [66]. So far, hydrazine is still the most widely used agent because of its strong reduction activity for removing oxygenated groups. However, chemical reduction of GO to rGO cannot fully remove oxygenated groups. Accordingly, rGO still contains a certain amount of residual oxygen groups. Alternatively, rapid heating of GO to 1050 °C under vacuum or inert atmosphere to generate TRG, such that oxygenated functional groups are removed as carbon dioxide [67].

### 2.5. Graphene-Polymer Nanocomposites

Pure graphene exhibits exceptionally high elastic modulus of about 1 TPa and intrinsic strength of 130 GPa [8], superior optical transparency of 97.7% [9], and excellent electrical conductivity and mobility of 2 × 10^5^ cm^2^ V^−1^ s^−1^ [68]. In this respect, graphene is an attractive filler material for polymers to form functional polymer nanocomposites [69]. The performance of polymers with high flexibility can be tailored for specific applications by adding fillers of micro- or nanoscale sizes [70]. Polymer composites inherit advantageous properties of their constituent components, and, what is more, polymers provide a protective matrix for embedded fillers from the mechanical damage. Conventional polymer microcomposites are commonly used as structural components in biomedical and industrial sectors due to their light weight, ease of fabrication, and low cost [71,72,73,74,75,76]. However, large volume filler contents (≥30%) are needed for achieving desired biological, mechanical, and physical behaviors. Large filler volume contents have adverse effects on the properties of polymer microcomposites. In this respect, graphene-based nanomaterials at low filler loadings can be used to fill and reinforce polymers.

As aforementioned, pristine CVD graphene is mainly developed for optoelectronic devices, touch screens, and solar photovoltaics. LPE-graphene flakes contain residual solvents and surfactants that have adverse health effects. In this respect, GOs with oxygenated groups are considered as ideal fillers for reinforcing polymers. Those functional groups render GO hydrophilic, thus can react with water-soluble polymer, such as polyvinyl alcohol [77]. From the literature, the elastic modulus of GO monolayer is 207.6 ± 23.4 GPa [78], being a quarter of the modulus of pure graphene. Nevertheless, the modulus of GO is still much higher than that of biopolymers, such as polylactic acid (PLA) with a modulus of about 2.7–3 GPa and polycaprolactone (PCL) of 0.4 GPa. So, GO can be used to reinforce GO/PLA and GO/PCL nanocomposites with enhanced mechanical performance, thermal stability, and biocompatibility. These polymer biocomposites can be prepared by several routes, including solution mixing, melt mixing, and electrospinning [79,80,81].

## 3. Cell Viability and Toxicity

Graphene-based nanomaterials can be either biocompatible or toxic to biological cells. The response of living cells to these nanomaterials depends greatly on their layer number, lateral size, purity, dose, surface chemistry, and hydrophilicity. The surface chemistries of graphene nanomaterials vary greatly because of different strategies adopted for their synthesis, and the availability of different molecules or polymers for surface functionalization. Generally, several major cell lines are employed for in vitro evaluation of nanomaterial toxicity, including phagocytes (e.g., macrophages) and non-phagocytic cells (e.g., endothelial and epithelial cells, cancer cells, erythrocytes, etc.). Proper understanding of how graphene nanomaterials interact with those cells is of crucial importance in using them for medical applications.

The interactions of graphene nanomaterials with cell membranes can also lead to the membrane damage and cytotoxicity. Phospholipids of cell membranes consist of a phosphate head group and two fatty acid chains. The primary head groups include choline, serine, glycerol, ethanolamine, inositol, and phosphatic acid. The various head groups in phospholipids confer them with distinctive properties. Furthermore, cell membranes also contain cholesterol molecules that play important roles in stabilizing membrane structure, maintaining fluidity, and modulating activities of membrane associated proteins. Pure graphene with no charges on the basal plane cannot interact electrostatically with phospholipids, but favoring hydrophobic interactions with the lipid tails. Moreover, hydrophobic interactions between pure graphene and cholesterol tail can extract or remove cholesterol molecules from the membrane, leading to the membrane damage. For instance, Bernabo et al. indicated that GO can interact with the cell membrane of swine spermatozoa, resulting in cholesterol extraction from the membrane [82]. From the theoretical simulation of biomembrane systems, cholesterol molecule extraction induces void formation and membrane deformation due to strong forces dragging from the graphene sheet, causing a loss of membrane integrity [83]. More recently, Duan et al. reported that GO can extract phospholipids from the cell membranes of human alveolar epithelial A549 and mouse macrophage Raw264.7 cells, producing surface pores, as evidenced by the SEM micrographs [84]. This effect reduced cell viability, leading to final cell death. They attributed this to the strong interactions between hydrophobic domains of GOs and lipid-tail carbons on the basis of molecular dynamics (MD) simulations.

The surface charge and surface chemistry of GO exert significant effects on the cellular interactions. GO has a high negative charge density conferred by oxygenated functional groups, thus possible electrostatic interactions between GO and lipids of the membrane may occur. In this context, Li et al. prepared five lipids with the same 18-carbon alkyl chain but different head group charges, and studied GO-lipid interactions using Langmuir monolayer technique [85]. They found that GO can interact with positively charged lipid head groups through electrostatic interactions, but not with neutrally or negatively charged lipids. As is known, the electrical charges of phospholipids of mammalian cell membranes are negative or neutral. It is unlikely that negatively charged lipids would attract GO of the same charges. Hu et al. indicated that negatively charged GO undergo electrostatic repulsion with lipids of the same charges, while hydrophobic interactions between negatively charged GO and lipids facilitate GO adsorption on the lipids [86]. These findings indicate that GOs can directly induce cell membrane damages through hydrophobic interactions without penetrating the cells. Very recently, Xia and coworkers investigated the effect of GO surface chemistry on the lipid membrane interactions using pristine GO, hydrated GO (hGO), and rGO [87]. They reported that hGO can induce lipid peroxidation of the surface membrane, leading to membrane lysis and the destruction of cell integrity. hGO was synthesized by reacting GO with sodium hydroxide solution at 50 or 100 °C. During the hydration process, epoxy rings of GO reacted with nucleophiles in the solution, creating C-OH groups and carbon radicals (*C), as detected by the electron paramagnetic resonance technique. These radicals were reactive due to the presence of unpaired electrons that reacted readily with oxygen to yield superoxide radicals, having capability of oxidizing unsaturated lipids and thiol groups on proteins to form lipoperoxides. This led to a failure in membrane integrity and cell death in human leukemic monocyte THP-1 and human bronchial epithelium BEAS-2B cells. The epoxy group of pure GO could also create carbon radicals, but to a lesser extent than hGO. As such, the cytotoxicity induced by the GO-based materials in both cell types is in the order hGO > GO > rGO.

Apart from the lipid membrane-nanomaterial interactions, graphene based nanomaterials can also enter cytoplasm because of their small size and sharp edges. They can penetrate cell membrane easily leading to membrane damage and leakage of cytoplasmic contents. Upon residing in living cells, they can induce toxicity through the creation of reactive oxygen species (ROS), which may cause mitochondrial disorder by reducing mitochondrial membrane potential (MMP), and cell membrane damage via the release of lactate dehydrogenase (LDH). In the latter case, ROS can induce lipid peroxidation by reacting with unsaturated fatty acids of membrane lipids to yield lipid peroxides, such as malondialdehyde (MDA). From these results, GO can induce extracellular and intracellular ROS generation, even at low doses, under a dose- and time-dependent manner. Therefore, ROS production, mitochondrial dysfunction and LDH leakage are the key factors relating to cell death as in the case of human HaCaT skin keratinocytes [88]. If the nanomaterials can enter the nucleus, they could interact directly with DNA and cause genotoxicity. Furthermore, in vitro and in vivo conditions, such as graphene dose, time of exposure, cell or animal type, and the technique used to assess cell viability can influence biocompatibility and toxic effect of graphene nanomaterials. Therefore, proper understanding of the interactions of graphene-based nanomaterials with biological systems and their adverse effects is necessary for further development and safe use of these nanomaterials.

### 3.1. In Vitro Cell Cultivation

#### 3.1.1. CVD-Grown Graphene

The use of pristine graphene provides us with a better understanding of direct response and interaction between nanostructured graphene and biological cells. From literature, conflicting results have been reported relating the effects of pure graphene films on the biocompatibility and cytotoxicity of cellular systems [89,90,91,92,93,94,95]. Zhang et al. exposed CVD-grown graphene with doses of 0.01, 0.1, 1, 10, and 100 µg/mL to neuronal PC12 cells for 1, 4, and 24 h [89]. At lowest dose of 0.01 µg/mL, graphene had no effect on metabolic activity, LDH release, and ROS. At a high dose of 10 µg/mL, graphene induced cytotoxic effects by decreasing mitochondrial activity based on the 3-(4,5-dimethylthiazol-2-yl)-2,5-diphenyltetrazolium bromide (MTT) assay results, while increasing ROS, caspase-3 and LDH levels. From the caspase 3 (apoptosis marker) test, apoptosis occurred at graphene doses ≥10 µg/mL. Thus, cytotoxic effects induced by graphene were dose- and time-dependent. It is generally known that ROS can oxidize cellular lipid, protein, and DNA molecules, thereby influencing cellular signaling and metabolism. Increased ROS production can cause mitochondrial membrane depolarization and promote caspase activation. Mitochondrial membrane depolarization results from a loss in mitochondrial membrane integrity, resulting in a decrease of MMP. This in turn retards ATP synthesis from mitochondrion, which is the power house of the cell. Caspases are intercellular proteases that are responsible for proteolytic cleavage. Caspase-3 is the main caspase that is involved in the execution of apoptosis.

On the contrary, Nayak et al. found that CVD-grown graphene films do not hamper the proliferation of human mesenchymal stem cells (hMSCs), but accelerates their specific differentiation into bone cells [90]. They investigated the effect of graphene on hMSCs proliferation employing four substrate materials with different stiffness and surface roughness, including polydimethylsiloxane (PDMS), PET, glass slide, and SiO_2_/Si. All graphene coated substrate specimens display a large increase in calcium deposits due to the bone formation. Furthermore, MTT assay results and immunofluorescence images reveal good cell viability and morphology. Kim et al. also demonstrated that CVD-grown graphene films exhibit stable cell attachment and excellent biocompatibility with primary adult cardiac cells (cardiomyocytes) [91]. Graphene films with superior electrical conductivity are particularly suitable for neural cell adhesion since the functions of neural cells are associated with electrical activities. In this respect, Park et al. reported enhanced the differentiation of human neural stem cells into neurons on the graphene coated glass substrate [92].

**Figure 5:**
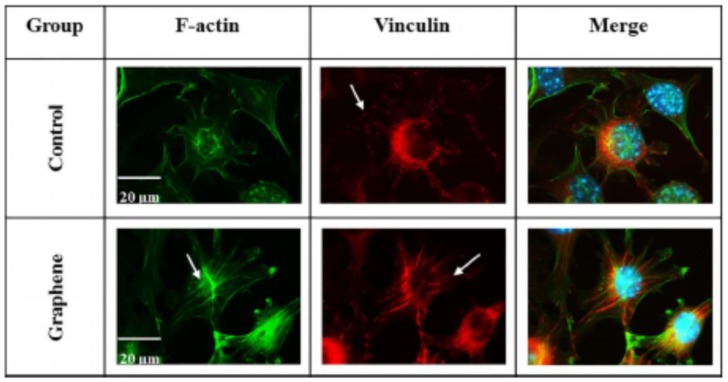
Immunolabeling of L929 cells grown for 24 h on the glass (control) and pristine graphene monolayer: phalloidine-FITC for actin cytoskeleton (green), anti-vinculin antibody for vinculin (red), and Hoechst 33342 stain for DNA (blue). White arrows indicate actin stress fibers (left panel) and focal adhesion protein—vinculin (middle panel). Reproduced from [95] with permission of Elsevier.

**Figure 6:**
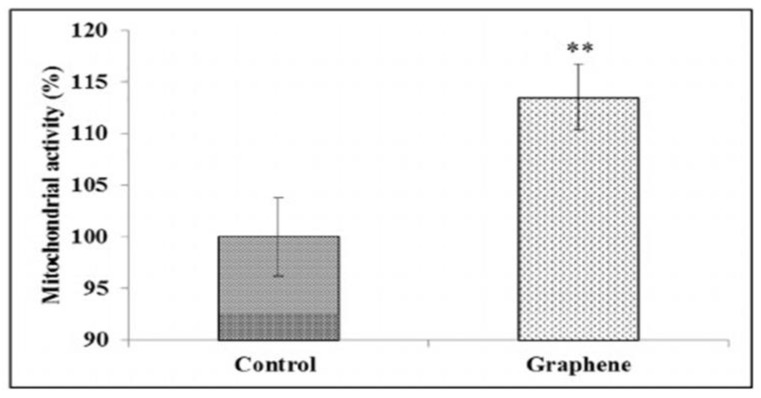
Mitochondrial activity (CCK-8 assay) of L929 fibroblasts grown on the glass and graphene substrates. Data are represented as mean ± SD (standard deviation) of three independent experiments. ** *p* < 0.01. Reproduced from [95] with permission of Elsevier.

Cell migration and adhesion is coordinated by large protein complexes generally known as the focal adhesions (FAs). FAs contain high levels of vinculin, talin, paxillin, α-actinin, focal adhesion kinase, etc. The protein vinculin in particular controls the cell adhesion [93]. It links actin filaments to the extracellular matrix (ECM), through talin and transmembrane protein integrins. Integrins play a key role in converging signals from the cell membrane to the inside of the cell. It is considered that the nanoscale topography of artificial substrates can regulate FAs and their associated cell adhesion, proliferation and differentiation [94]. Very recently, Lasocka et al. studied the adhesion and proliferation of L929 mouse fibroblasts on CVD-grown graphene film [95]. They indicated that graphene film exhibits no cytotoxicity to L929 fibroblasts. Furthermore, nanostructured graphene film increases cell adhesion at 24 h cultivation (Figure 5). From immunofluorescent images, vinculin and actin stress fibers can be observed on fibroblasts growing on both graphene film and glass substrate. The cells on graphene film exhibit a stronger expression of vinculin in terms of number of focal adhesions. In particular, actin stress fibers are more apparent and evident in fibroblasts growing on graphene. Stress fibers are generally composed of bundles of F-actin (cytoskeleton protein) filaments, which are crosslinked together by α-actinin. It appears that nanostructured graphene promotes the expression of FAs by regulating vinculin expression, thus enhancing cell adhesion. Lasocka et al. also employed CCK-8 colorimetric assay for assessing viable cell and proliferation, and found that the mitochondrial activity of L929 was about 12% higher than that on a control glass specimen (Figure 6). CCK-8 contains a tetrazolium salt, WST-8, comprising of [2-(2-methoxy-4-nitrophenyl)-3-(4-nitrophenyl)-5-(2,4-disulfophenyl)-2H–tetrazolium, monosodium salt], which is reduced to water soluble formazan by cellular dehydrogenases. The amount of formazan produced is directly proportional to the number of living cells. The detection sensitivity of CCK-8 is higher than the other tetrazolium salts, such as MTT and WST-1.

**Figure 7:**
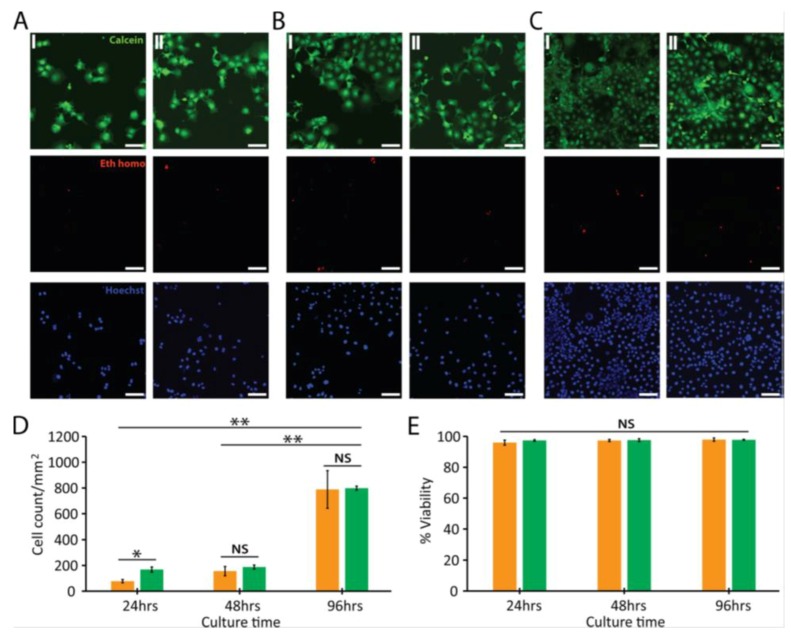
Live−dead assay for Cos-7 cells cultivated on (**I**) glass and (**II**) graphene for (**A**) 24 h, (**B**) 48 h, and (**C**) 96 h. Green, red, and blue denote live cells, dead cells, and cell nuclei, respectively. Scale bars: 100 μm. (**D**) cell number and (**E**) % viability of Cos-7 cells cultivated on the glass (orange) and graphene (green) for 24, 48, and 96 h, respectively. * and ** denote *p* < 0.05 and *p* < 0.005, respectively. NS implies no statistically significant difference. Reproduced from [96] with permission of the American Chemical Society.

More recently, Rastogi et al. studied the effect of LPCVD-grown graphene films on the viability and cell stress of both nonneuronal (monkey renal fibroblast; Cos-7) and neuronal (rat hippocampal neuron) cells [96]. They reported that graphene enhances cell adhesion and the growth of both cell lines. In addition, graphene exhibits no detrimental effect on the MMP and morphology of both cell types, demonstrating that pristine graphene does not induce cell stress. Live-dead assay and tetramethylrhodamine ethyl ester (TMRE) assay were adopted in their study. TMRE is a quantitative fluorescence marker for mitochondrial activity. Live-dead assay is a fluorescent cell viability test for assessing live and dead cells based on the detection of membrane integrity and cytotoxic consequences. The membranes of viable cells are intact and tight, but dead cell membranes are disrupted or damaged. The test employs calcein acetoxymethyl (Calcein-AM) and ethidium homodimer dyes for staining live and dead cells, respectively. Calcein-AM stains live cells green, while EthD-III stains dead cells red. Calcein AM is a non-fluorescent compound and it is converted to a green fluorescent calcein due to the hydrolysis reaction by intracellular esterases in live cells. Figure 7 shows live-dead assay results for Cos-7 cells cultured on pristine graphene and glass (control) for different periods. Apparently, graphene films exhibit no detectable cytotoxic effects on cell viability. The films promote cell adhesion and growth, especially at 96 h (Figure 7C (II)).

In recent years, titanium and its alloys have increasingly been used for making dental implants. Ti-based alloys generally exhibit much higher corrosion resistance than stainless alloys [97,98]. However, Ti-based metals suffer from high wear loss during their life service inside the oral cavity. Surface modification of dental implants with hard coatings is known to be very effective to combat wear issue and bacterial dental plaque accumulation on the implants. In this respect, inert graphene film with high hardness is an attractive material for coating dental implants. So, as-synthesized CVD-graphene film can be transferred onto Ti metal substrate to improve its wear resistance and bactericidal property. Zhou and coworkers investigated the adhesion, proliferation, and osteogenic differentiation of human adipose-derived stem cells (hASCs) and human mesenchymal stem cells (hMSCs) in vitro and in vivo when exposed to CVD-graphene covered Ti discs [99,100]. For the in vivo test, CVD-graphene/Ti discs were implanted into the back subcutaneous area of nude mice. Their results indicated that pristine graphene promotes osteogenic differentiation of hASCs and hMSCs in vitro and in vivo.

#### 3.1.2. Graphene Oxide and Its Derivatives

##### Graphene Oxide

Extensive studies have been conducted on the biocompatibility/cytotoxicity of GOs due to their ease of fabrication and relatively low cost. GO can enhance cell viability and cause cell death depending on the size, dosage, time, cell type, and surface chemistry. Because of the different surface oxidation states and features between GO, rGO, and TRG, such graphene materials have distinct chemical and physical properties. GO possesses many defects, such as vacancies due to synthesis, as revealed by high-resolution TEM images and Raman spectra [31,32]. TRG produced from rapid heating of GO at high temperatures exhibits a wrinkled feature [67]. Modified Hummers’ process is commonly used by the researchers for oxidizing graphite. However, various oxidation times and temperatures, different types and concentrations of oxidants have been employed for synthesizing GOs [59,60,61]. Consequently, the resulting GOs contain different O contents or O/C ratios. The O/C ratios in GOs affect their structural properties greatly, as manifested in the degree of exfoliation, number of graphene layer, thickness and lateral layer size, defective fraction, functional group concentration, etc. As a result, GOs with varying levels of oxidation and impurities would interact differently upon exposure to the same cell line. The structural diversity of GOs leads to conflicting results in the literature relating to biocompatibility and toxicity. Therefore, it seems necessary to standardize the synthesis process in using GOs for biomedical applications.

The synthesis of GO generally involves the use of several strong oxidizers that can introduce residual impurities or contaminants in the final products. Wong et al. determined the impurity types and contents in both GO and rGO. They reported that chemical oxidation and reduction steps during fabrication can introduce various metallic elements into GO and rGO [101]. The main impurities were manganese (about 2,290 ppm), potassium (820 ppm), sodium (96 ppm), and Fe (82 ppm). These impurities came from sulfuric acid, sodium nitrate, and potassium permanganate reagents. Other detectable metal impurities included Ca, Co, Cr, Cu, Ni, Pb, and Zn. Furthermore, hydrazine and sodium borohydride (NaBH_4_) reducing agents led to higher Fe and Na concentrations in rGO. Fe and Na levels increased from 82 to 123 and 7600 ppm, respectively, by reducing with NaBH_4_. Residual Mn and Fe impurities in GOs affected their physical properties markedly [101].

Heavy metals, such as Mn, Fe, and Cr are particularly harmful or toxic to mammalian cells. Manganese can induce toxicity in pulmonary and nervous cells [102,103], and cause mitochondrial disorders by decreasing MMP and inhibiting Ca^2+^ activation through control of the rate of ATP synthesis during oxidative phosphorylation. As such, the presence of Mn in the nucleus of PC12 cells can lead to chromatin condensation, DNA fragmentation, and increased mRNA levels of caspase-3 [93]. Mn can also cross the blood-brain barrier and enter the brain. Upon exposure to human body, manganese toxic effects are found mainly in the respiratory tract and brain tissues, including lung embolism, bronchitis, and nerve damage [103]. It has been reported that single-walled carbon nanotubes with trace amounts of Co, Cr, Fe, Ca, and Si are more toxic than impurity-free GO (synthesized from electrochemical route) exposed in the same cell lines [104]. It is noteworthy that chemical agents, such as anhydrous hydrazine, hydrazine monohydrate, and sodium borohydride that are typically employed for reducing GO are highly toxic to humans. These chemicals must be avoided for preparing rGO used for the cell manipulation. Instead, environmental friendly natural products, such as glucose, tea polyphenol, and ascobic acid can be used as reducing agents [65]. In recent years, efforts have been made to synthesize rGO by using environmental friendly extracts derived from the natural plants, such as mushroom, palm leaf, and aloe vera [105,106]. For instance, Dasgupta et al. successfully reduced GO in aqueous solution with polysaccharide extracting from a wild edible mushroom [105]. They reported that rGO was biologically safe at concentrations below 100 μg/mL.

**Figure 8:**
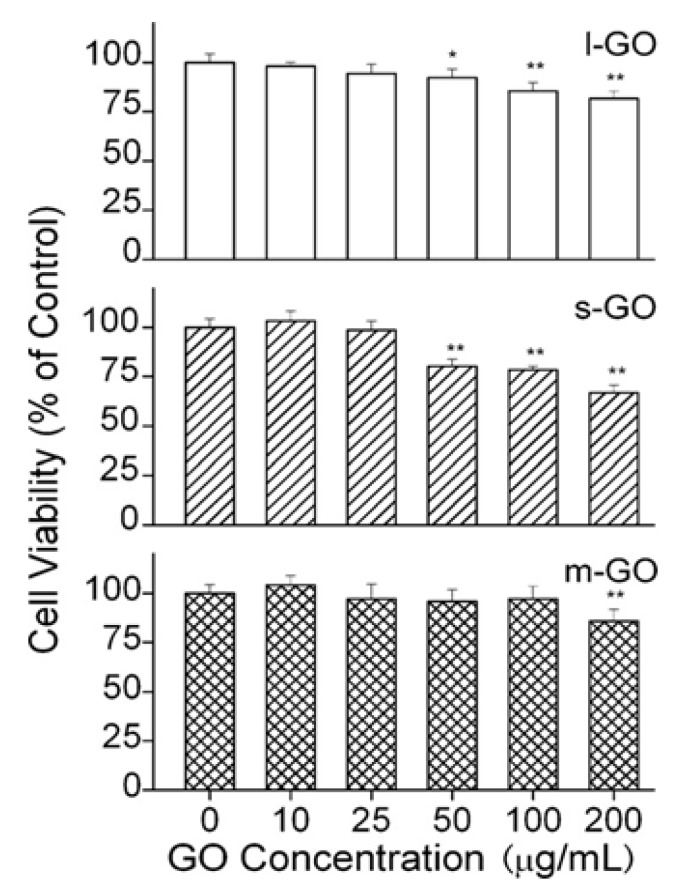
The effects of graphene oxide (GO) doses and sizes on the viability of A549 cells. Reproduced from [46] with permission of Elsevier. * and ** denote *p* < 0.05 and *p* < 0.01 vs. the control, respectively.

**Figure 9:**
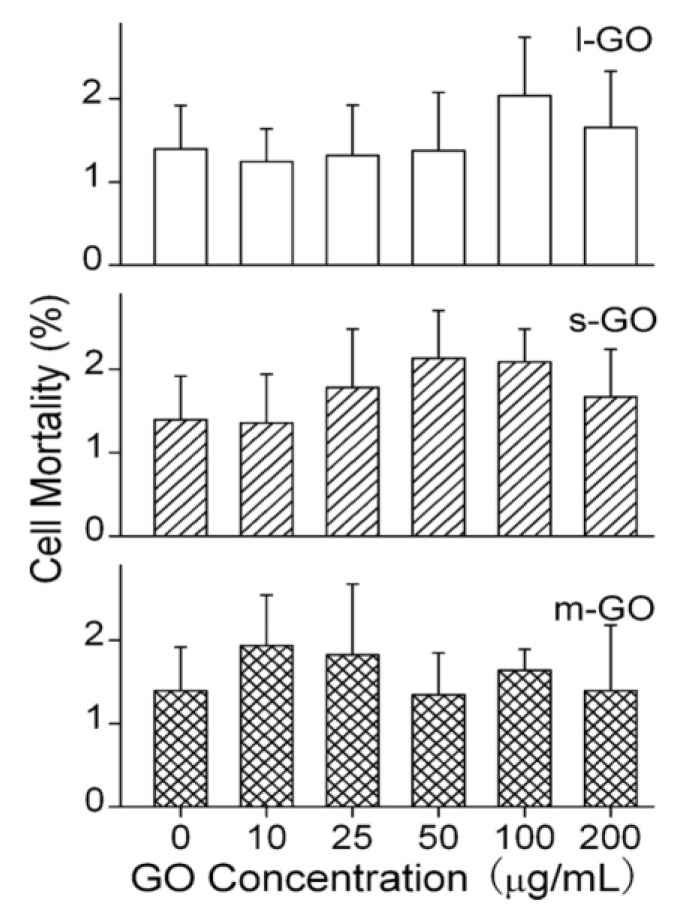
The effects of GO doses and sizes on the mortality of A549 cells. Reproduced from [46] with permission of Elsevier.

Chang et al. studied the effect of size and dose on the biocompatibility of GO exposed to A549 cells [46]. GOs with sizes of 780 ± 410 nm (*l*-GO), 430 ± 300 nm (*m*-GO), and 160 ± 90 nm (*s*-GO) were used in their work. GO showed good biocompatibility with A549 cells and exhibited no signs of cytotoxicity (Figure 8). CCK-8 assay showed that the effect of *l*-GO and *m*-GO on the viability of A549 cells was small. The cells maintained a high level of viability (over 80%), even at high GO concentration of 200 µg/mL. However, viability loss was observed for *s*-GO at 200 µg/mL. At this dosage, cell viability was 67% for 24 h incubation. Moreover, Trypan blue exclusion assay, in which the dead cells were dyed in blue and live cell remained unchanged was employed to assess cell mortality. Live cells generally exhibit intact cell membranes; trypan blue cannot pass through their membranes and enter the cytoplasm. On the contrary, the dye penetrates the porous cell membrane and it enters the cytoplasm of dead cells. Although *s*-GO induced viability loss at 200 µg/mL, no mortality increase of A549 cells is found. The mortality maintains at 1.5%, being similar to that of the control (1.4%) (Figure 9). Using TEM, no cellular uptake of GO by the A549 cells is found as well. Similarly, Bengtson et al. also reported that GO and rGO of different sizes do not induce toxicity in murine lung epithelial cell line [44].

From the literature, GOs exhibit dose- and size-dependent toxicity towards different cell lines, e.g., human fibroblast, human hepatocellular carcinoma, human skin keratinocyte, etc. [43,47,87,88]. Wang et al. demonstrated that GOs with doses <20 μg/mL have no toxic effect, but doses >50 μg/mL causes cytotoxicity by inducing cell apoptosis. They attributed cytotoxicity to the penetration of GOs into lysosomes, mitochondrion, endoplasm, and cell nucleus [47]. Lammel et al. studied the effect of human hepatocellular carcinoma cell (HepG2) exposure to GOs, with doses of 1, 2, 4, 8, and 16 µg/mL [43]. Dose- and time-dependent cytotoxicity was found in the HepG2 cells, even at low concentration of 4 µg/mL. The GO-induced toxicity aroused from a loss of plasma membrane structural integrity and a decrease in MMP, owing to a strong physical interaction of GO with phospholipid bilayer. GO then penetrated through the plasma membrane into the cytosol, generating ROS and causing an augmented number of apoptotic cells. Very recently, Bernabo et al. assessed the viability of swine spermatozoa by exposing them to 0.5–50 µg/mL GOs for 1 to 4 h [82]. Cell death occurred at GO dosages ≥10 µg/mL after 1 h exposure. At lower GO doses, cholesterol was extracted from the sperm cell membrane, leading to the membrane damage.

**Figure 10:**
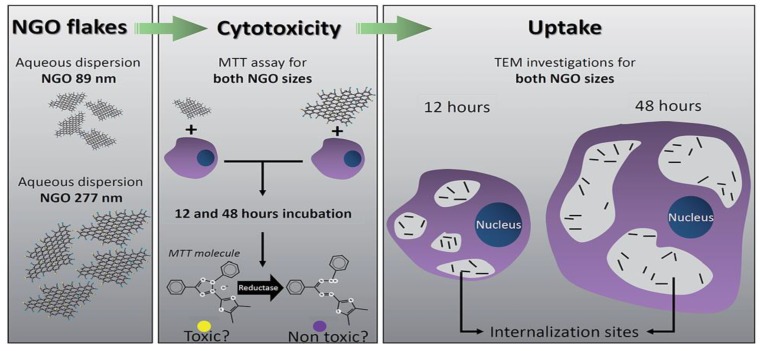
Schematic showing the strategies used to assess size-dependent cytotoxicity and internalization of nanographene oxide (NGO). Reproduced from [111] with permission of the Royal Society of Chemistry.

**Figure 11:**
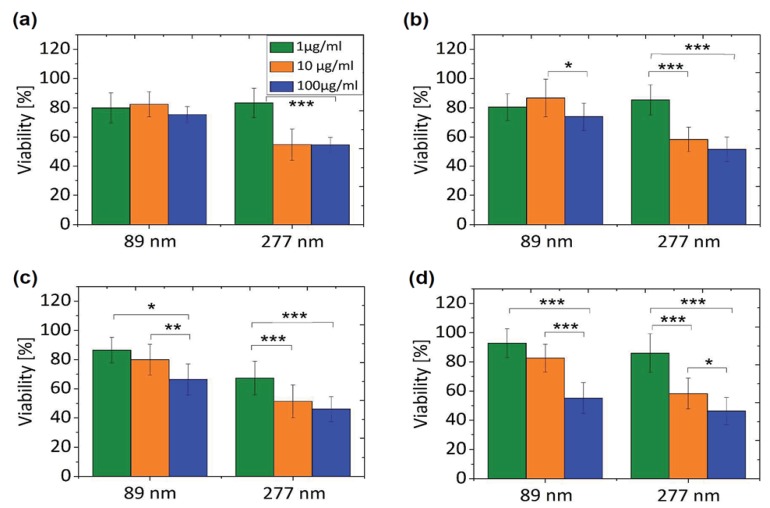
(**a**,**c**) are 3-(4,5-dimethylthiazol-2-yl)-2,5-diphenyltetrazolium bromide (MTT) results of HeLa cells cultivated with GOs of different sizes and doses for 12 h and 48 h, respectively. (**b**,**d**) are MTT results of macrophages cultivated with GOs of different sizes and doses for 12 h and 48 h, respectively. * *p* < 0.05; ** *p* < 0.01; *** *p* < 0.001; bars without mark mean a non-significant *p* > 0.05. Reproduced from [111] with permission of the Royal Society of Chemistry.

**Figure 12:**
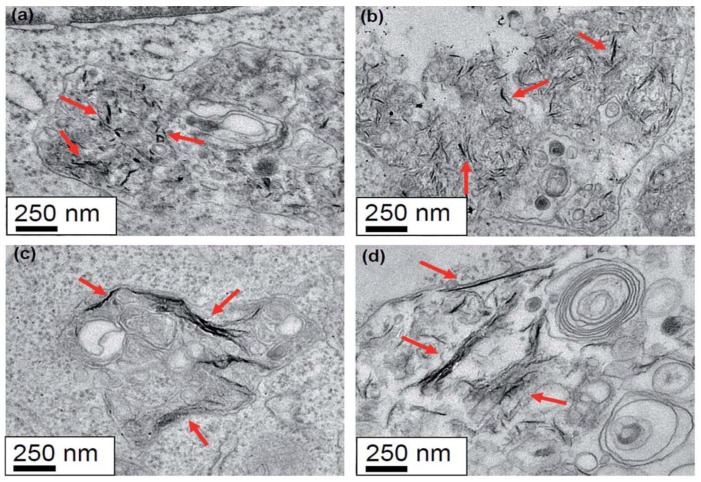
(**a**,**b**) are TEM images of a macrophage cultivated with small GOs for 12 and 48 h, respectively. (**c**,**d**) are TEM images of a macrophage cultured with large GOs for 12 and 48 h, respectively. GO flakes with sizes of 89 and 277 nm are internalized by a macrophage, as indicated by the arrows. Reproduced from [111] with permission of the Royal Society of Chemistry.

The effects of GOs on the biocompatibility/toxicity of immune cells are now considered. The interactions of GOs with the immune and circulatory systems are crucial; once administrated, they directly come in contact with such systems [107,108]. Macrophages are the cells involved in the defense mechanism of innate immune system and provide a first line of defense against microorganisms. Macrophages originate from bone marrow monocytes that migrate through the blood circulation. In the presence of bacteria and foreign objects, activated macrophages secrete cytokines and chemokines to attract cells with chemokine receptors, such as neutrophils and monocytes from the bloodstream. Several studies have been conducted on the macrophages acting as the target cells for GOs [109,110,111]. Pristine graphene and GOs induce the formation of several proinflammatory cytokines, including IL-1α, IL-6, IL-10, and tumor necrosis factor alpha (TNF-α), and chemokines, such as monocyte chemoattractant protein-1 (MCP-1), macrophage inflammatory protein-1α (MIP-1α), and MIP-1β upon macrophage activation [107]. Macrophages are well known to exhibit phagocytic behavior, leading to internalization mechanism. In this respect, GOs can be internalized by macrophages, resulting in inflammation. Russier et al. reported that the phagocytic ability of macrophages depends on the size of GO flakes [109]. Macrophages have the greater ability to internalize smaller GOs than large ones. Similarly, Ma et al. demonstrated that small GOs with lateral sizes of 50–350 nm are easily internalized by murine J774A.1 macrophages, while large GOs (750–1300 nm) are preferentially adsorbed on their plasma membranes, thereby triggering toll-like receptor (TRL) and nuclear factor NF-κB pathways to promote pro-inflammatory [110]. Large GOs are more likely to interact with the cell membrane due to their large surface areas. Very recently, Mendes et al. investigated the effect of the size of GOs on the viability/toxicity of macrophages and cervical cancer HeLa cells (Figure 10) [111]. GOs with an average size of 89 and 277 nm were used in their study. They found that large GO flakes induce higher levels of toxicity than small flakes, especially at a longer incubation time of 48 h (Figure 11). TEM examination revealed that GOs of different sizes are internalized by the macrophage (Figure 12) and HeLa cells at 12 and 48 h incubation. In particular, macrophages experienced morphological change for phagocytosis after cultivation for 48 h, regardless of the sizes. This morphological change led to the formation of large vacuoles containing GOs.

As aforementioned, GOs can interact with cellular membranes and they induce damages through the formation of ROS, the extraction of membrane lipids or cholesterols, and the generation of carbon radicals. Red blood cell (erythrocyte) generally lacks repair capability due to the absence of a nucleus and mitochondria. The membrane damage of erythrocyte is termed as the hemolysis. Xia and coworkers indicated that lipid peroxidation could lead to a failure in the membrane integrity of murine erythrocytes. Hydrated GO induced severe hemolysis than GO and rGO [87]. Liao et al. reported that GOs (765 ± 19 nm) activated hemolytic activity of human erythrocytes. Sonicated GOs with a smaller size (342 ± 17 nm) exhibited a higher hemolysis rate than large GOs [45]. However, coating GOs with chitosan nearly eliminated hemolytic activity. Thus, the interaction modes of GOs with the cells (i.e., suspension vs. adherent cells) affect the viability of erythrocytes. Recently, Papi et al. also indicated that the hemolytic activity of human erythrocytes increases with reducing GO sizes. This activity was negligible when the plasma protein corona was adsorbed on the GO surfaces [112].

**Figure 13:**
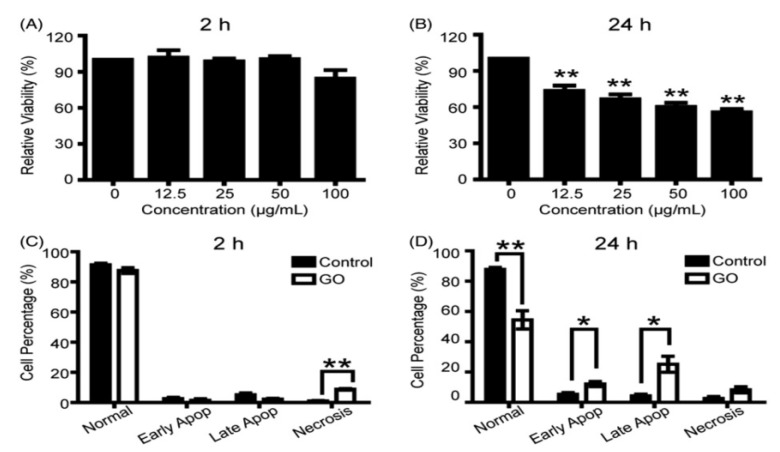
WST-8 assay results for human corneal epithelium cells (hCorECs) exposed to GOs of various doses for (**A**) 2 h and (**B**) 24 h, respectively. Apoptosis flow cytometric analysis of hCorECs exposed to GO (50 µg/mL) for (**C**) 2 h and (**D**) 24 h, respectively. Three independent experiments were performed for both assays. Data were presented as mean ± SEM (standard error of mean). * denotes *p* < 0.05 and ** represents *p* < 0.01. Reproduced from [113] with permission of Taylor & Francis.

**Figure 14:**
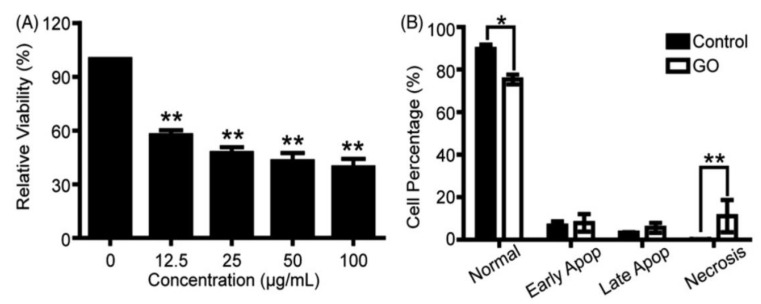
(**A**) WST-8 assay for hConECs exposed to GOs of different doses for 24 h. (**B**) Apoptosis flow cytometric analysis of hConECs exposed to GO (50 µg/mL) for 24 h. Three independent experiments were performed for both assays. Data were presented as mean ± SEM. * denotes *p* < 0.05 and ** represents *p* < 0.01. Reproduced from [113] with permission of Taylor & Francis.

Apart from the GO-erythrocyte interactions, little information is available in the literature relating the toxic effect of GOs on human primary cells. Primary cells are directly obtained from living tissue and established for growth in vitro. They mimic the physiological state of cells in vivo and produce more useful results representing human body system. Ethical permission for the acquisition of primary human cells from healthy and diseased tissue organs is needed. Very recently, Wu et al. assessed the toxicity of GOs upon exposure to primary human corneal epithelium cells (hCorECs) and human conjunctiva epithelium cells (hConECs) with doses of 12.5–100 µg/mL [113]. From the WST-8 assay data, GOs do not induce cytotoxicity to hCorECs at 2 h (Figure 13A), with the exception of increased necrosis on the basis of flow cytometry-apoptosis detection at 50 µg/mL (Figure 13C). However, GOs cause significant cytotoxicity to hCorECs after 24 h. The cell viability decreases markedly with increasing GO doses (Figure 13B). At 50 µg/mL GO, flow cytometry reveals a significant increase in apoptotic cells (Figure 13D). In the case of hConECs, the WST-8 assay indicates that nearly 40% of hConECs are dead by cultivating with 12.5 µg/mL GO for 24 h. Cell viability is only 50% as the GO concentration increases to 100 µg/mL (Figure 14A). At 50 µg/mL GO, flow cytometry indicates that the percentage of normal cells drops significantly, whereas the percentage of necrosis cells rises markedly after 24 h exposure (Figure 14B). They concluded that GOs induce dose- and time-dependent cytotoxicity in both hCorECs and hConECs through the oxidative stress generation from the ROS detection.

##### PEGylation

Graphene oxides can be functionalized with a variety of molecules and biomolecules to increase their dispersibility in physiological solutions, thus improving their biocompatibility. GOs are typically functionalized with PEG, amine, acrylic acid, and dextran. In particular, GO-PEG has been shown to exhibit good biocompatibility as compared to GO [48,114,115]. Wojtoniszak et al. reported that PEGylated GO shows the best biocompatibility with mice fibroblast cell line (L929) under dosages of 3.125–12.5 µg/mL [114]. The relative cell viability is over 80% at this dose range. By increasing the dose to 50 µg/mL, cell viability drops markedly to about 60%, i.e., about 40% cells are dead. Du et al. treated GO-PEG with lymphoma cells and studied cell viability using CCK-8 assay [115]. Cells that were treated with GO-PEG (10–100 μg/mL) for 24 h had viability rates over 80%, implying low cytotoxicity and high biocompatibility of PEG-GO. On the contrary, PEgylated GO sheets were reported to show cytotoxicity for some cell lines [116,117,118]. For instance, GO-PEG sheets were found to disrupt F-actin filaments of Saos-2 human osteosarcoma osteoblasts, MC3T3-E1 preosteoblasts, and RAW-264.7 macrophages after internalization. These filaments were needed for regulating cell migration. The disruption induced cell-cycle alterations, apoptosis, and oxidative stress in these cell lines [116]. Recently, Mendonca et al. indicated that rGO-PEG induces excess ROS in murine astrocytes at 100 µg/mL, reducing cell viability to only 16% after 24 exposure [117]. Luo et al. demonstrated that PEGylated GO sheets induced immune response in peritoneal macrophages through the secretion of cytokines for enhancing integrin beta-8 related signaling pathways [118]. As such, PEGylation did not act to passivate the surfaces of graphene-based materials.

**Figure 15:**
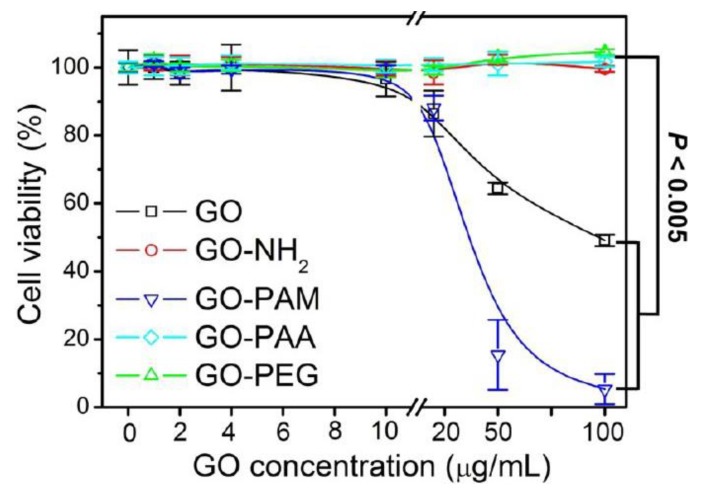
CCK-8 assay results of macrophage cells (J774A.1) treated with GO, GO-NH_2_, poly(acrylamide)-functionalized GO (GO-PAM), poly(acrylic acid)-functionalized GO (GO-PAA), and poly(acrylamide)-polyethylene glycol (GO-PEG) at different concentrations for 24 h (*n* = 5). Reproduced from [119] with permission from The American Chemical Society.

More recently, Xu et al. studied the viability of murine macrophages (J774A.1) treated with GO, GO-NH_2_, poly(acrylamide)-functionalized GO (GO-PAM), poly(acrylic acid)-functionalized GO (GO-PAA), and GO-PEG in vitro and in vivo [119]. Among these, GO-PEG and GO-PAA were found to be less toxic than GO, and GO-PAA exhibited the best compatibility in vitro (Figure 15) and in vivo. They attributed the variations to the differential compositions of protein corona, particularly immunoglobulin G (IgG), formed on their surfaces that dictate their cell membrane interactions and cellular uptakes. As known, proteins cover the surfaces of nanomaterials quickly when those materials come into contact with a physiological environment. Such adsorbed protein layer is usually termed as the protein corona. In a recent work carried out by Syama et al. [120], rGO-PEG (PrGO) was found to have no toxicity in mouse bone marrow mesenchymal stem cells (MSCs), and it did not appear to impair their differentiation. PrGO was prepared by reducing GO-PEG with sodium borohydride. PrGO was internalized by MSCs and distributed throughout the cytoplasm. Although it also induced ROS generation inside the cell, no change in cellular proliferation or function was detected. In another study, PrGO exhibited a dose dependent decrease in viability of A549 alveolar epithelial cells after 24 h exposure [121]. The doses ranged from 1–200 μg/mL. MTT assay revealed that more than 80% of the cells was viable at 10 μg/m, but the viability dropped to below 76% at 25 μg/mL after 24 h. Neural red uptake (NRU) assay also showed a similar decrease trend in dose-dependent cell viability. Moreover, PrGO induced a dose- and time-dependent increase in ROS, reduced MMP, and triggered inflammatory response upon internalization, leading to apoptosis. Hence, MSCs and A549 cell lines responded differently to PrGO prepared from the same procedures. In contrast, rGO was not internalized by A549 cells, but it adhered to the plasma membrane. As such, it activated NF-κB by binding to cell surface TLRs and triggered inflammatory response accordingly.

##### Reduced Graphene Oxide

Akhavan et al. prepared rGO nanoplatelets by sonication PEGylated GO sheets, followed by reducing in hydrazine and bovine serum albumin [122]. The cell viability test revealed significant cell destructions in hMSCs by treating with only 1.0 μg/mL rGO. The rGO nanoplatelets induced genotoxic effects on the cells through DNA fragmentation and chromosomal aberration. As mentioned previously, hydrazine is highly toxic and explosive, so a significant toxic effect may have come from residual hydrazine on the rGO surface. For biomedical applications, it deems necessary to consider the toxic effects of solvents and reducing agents employed for exfoliating the graphene sheets. Many of these chemical reagents do not meet the safety standards. Wu et al. recently demonstrated that rGO is more toxic than GO upon exposure to bone marrow-derived macrophages (BMDMs) and J774A.1 cell line [123]. In their study, rGO provoked higher levels of pro-inflammatory cytokines, i.e., TNF-α and IL-6 in the macrophages, particularly in BMDMs. They attributed this to the rGO induces higher oxidative stress in the macrophages compared to GO. It is noted that they employed sodium sulfide (Na_2_S) for reducing GO. Sodium sulfide is a toxic agent that causes serious human skin irritation or burns, and it reacts with moist air or acid to produce toxic H_2_S. We considered that residual Na_2_S in rGO induces pro-inflammatory cytokines, leading to the activation of macrophages.

**Figure 16:**
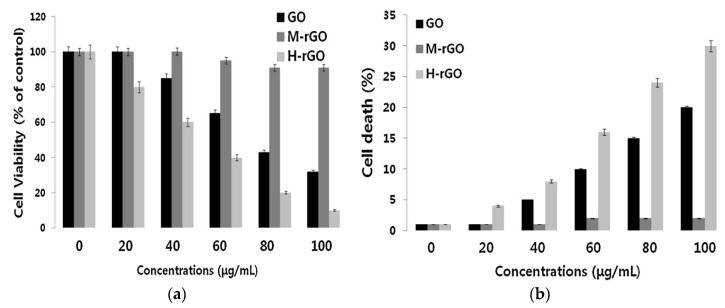
(**a**) WST-8 assay for primary mouse embryonic fibroblasts exposed to GO, M-rGO, and H-rGO of different concentrations for 24 h. Error bars represent standard error of the mean (*n* = 3); p < 0.05; (**b**) Cell mortality of primary mouse embryonic fibroblasts (PMEF) cells determined from trypan blue assay upon 24-h exposure to GO, M-rGO and H-rGO of different concentrations. Error bars represent standard error of the mean (*n* = 3); *p* < 0.05. Reproduced from [124] with permission of Elsevier.

To address the cytotoxicity induced by hydrazine, Gurunathan et al. successfully prepared rGO using a bacteria strain, i.e., Pseudomonas aeruginosa as a reducing reagent for GO [124]. They then exposed primary mouse embryonic fibroblasts (PMEFs) to pristine GO, microbially reduced GO (M-rGO), and hydrazine reduced GO (H-rGO) of different concentrations. WST-8 assay results indicated that the cell viability maintains at about 91.0 ± 2.0% by treating PMEFs with 20–100 μg/mL M-rGO (Figure 16a). However, H-rGO shows a significant level of toxicity comparing with M-rGO. The cell viability drops to below 60% by treating with 20 μg/mL H-rGO. Trypan blue assay data also revealed that the cell mortality maintains at about 4% by treating with high M-rGO level (100 μg/mL), being the same as that of the control (Figure 16b). In contrast, H-rGO induces the highest rate of death when comparing with M-rGO. Their results clearly demonstrate the toxic effect of hydrazine to biological cells. Although bacteria biomass can reduce GO and minimize the toxic effect of rGO effectively, their use is very limited for the biomedical field. This is because Pseudomonas aeruginosa can cause urinary tract and respiratory infections, pneumonia, and dermatitis diseases.

**Figure 17:**
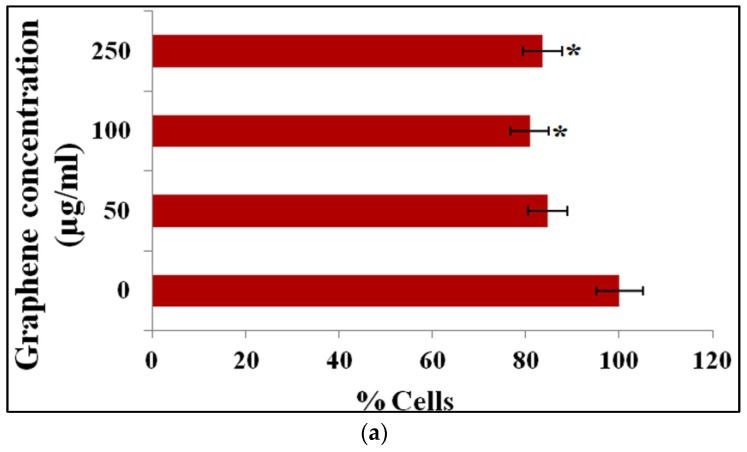

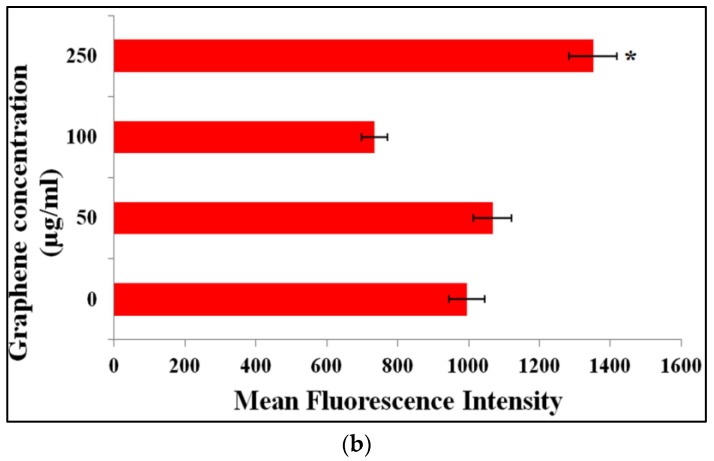
(**a**) Neural red uptake and (**b**) flow cytometry (PI estimation) results for green synthesized rGO exposed to human lymphocyte cells. Reproduced from [105] with permission of Public Library of Science.

More recently, Dasgupta et al. used green synthesized rGOs of different dosages (50, 100, and 250 µg/mL) for investigating their interactions with human lymphocytes derived from fresh blood samples [105]. GOs were reduced in a wild mushroom extract solution to yield rGOs. Several assays, including MTT, neural red uptake, flow cytometry, and ROS were used for in vitro cell tests. MTT data revealed no significant change in mitochondrial activity. Neutral red uptake (NRU) assay showed a loss of lysosomal integrity at rGO contents ≥100 μg/mL (Figure 17a). Flow cytometry indicated that propidium iodide (PI) uptake increases markedly at the highest rGO level (250 μg/mL), demonstrating a loss of membrane integrity (Figure 17b). Apparently, green synthesized rGOs are less toxic when comparing with rGO counterparts prepared from conventional strategy using toxic reducing reagents.

In a recent study conducted by Mittal et al., GO was found to induce toxicity at higher levels than rGO upon exposure to human alveolar epithelial A549 and bronchial epithelial (BEAS-2B) cells [125]. The presence of oxygenated groups on GOs led to the increase of cytotoxicity, owing to the ROS and oxidative stress formation. Similarly, Das et al. demonstrated that GO is more toxic than rGO of same size upon exposure to human umbilical vein endothelial cells (HUVEC) [126]. GO with abundant oxygen functional groups has a greater potential to interact with endothelial cells, leading to a high ROS level and DNA damage. The oxidative stress is manifested by elevated gene expressions of heme oxygenase 1 (HO1) and cytosolic thioredoxin reductase (TrxR).

**Figure 18:**
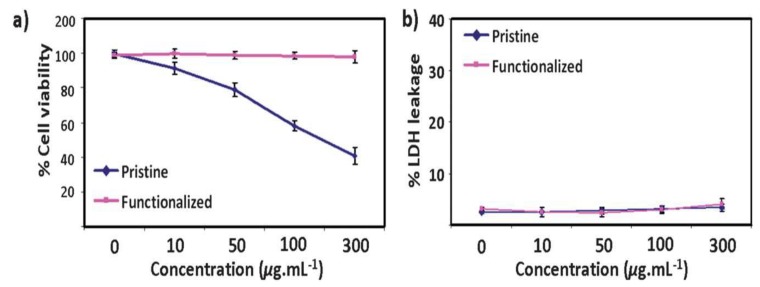
(**a**) Cell viability and (**b**) lactate dehydrogenase (LDH) leakage of Vero cells treated with TRG and f-TRG at different concentrations. Reproduced from [127] with permission of The Royal Society of Chemistry.

Sasidharan et al. studied the effect of TRG and carboxyl-functionalized TRG (f-TRG) on the viability of monkey Vero kidney cells [127]. Contact angle measurement revealed that TRG flakes were hydrophobic with a large water contact angle of 162°, and f-TRG flakes were hydrophilic with an angle of 30° due to the presence of carboxyl groups. Hydrophobic TRG flakes were found to be accumulated on the plasma membrane, whereas f-TRG flakes were internalized by the cells, as revealed by the fluorescence confocal microscopic images. As such, hydrophobic TRG may induce strong interactions with membrane lipids leading to direct physical toxicity, such as lipid extraction, as mentioned previously. The viability of cells decreased markedly with increasing TRG concentrations. At 100 mg/mL, nearly 50% cells were dead, and increased to about 60% at 300 mg/mL In contrast, f-TRG specimens exhibited negligible effects on the viability, even at a high concentration of 300 mg/mL (Figure 18a). Despite the fact that f-TRG flakes were internalized by the cells, their metabolic activity remained unaffected. Figure 18b shows cellular LDH leakage due to the TRG and f-TRG exposures. As is known, LDH molecules are released into the medium from the cells with damaged membrane. So, the LDH level in the culture medium is an indicator of cellular membrane damage. At 300 mg/mL, TRG flakes caused complete cell death but there was no LDH leakage. It is unclear how f-TRG could have a beneficial effect on cellular viability, and its carboxyl groups do not interact with Vero cells, as in the case of GO (with carboxyl groups) showing interactions with human epithelial lung cells and HUVEC. This effect may be due to different cell types and physiochemical characteristics of the GO and f-TRG. Table 1 summarizes the size-, dose-, and time- dependent toxicity induced by graphene and its derivatives upon exposure with mammalian cells.

### 3.2. In Vivo Animal Model

In the in vivo system, the whole living animal is employed to assess the effect of toxicity induced by graphene-based nanomaterials. Useful information relating cell-graphene interactions can be obtained by histological and microscopic examinations of relevant organ tissues and inflammatory cells of the animals after oral feeding, intravenous administration, intraperitoneal injection (*i.p.*), intratracheal instillation, oropharyngeal aspiration, subcutaneous injection, etc. The in vivo effect of GO and its derivatives depends greatly on the chemical nature of nanomaterials, exposure time, dose and administration route, and type of the animals employed for the tests. From the early study by Yang et al., PEG-GO did not induce appreciable toxicity in mice after intravenous administration under a dose of 20 mg/kg for three months based on blood biochemistry and hematological and histological examinations [48]. PEG-GO mainly accumulated in the liver and spleen, and it removed from these organs by renal and fecal excretion. In another study, they administrated 4 mg/kg GOs into balb/c mice through *i.p.* and oral administration followed by histological and hematological examinations [128]. According to their findings, GO retained in the mouse body for a long period of time following *i.p.* injection, its toxicity to the mice was insignificant. Ali-Boucetta et al. indicated that purified GO did not induce inflammation and granuloma formation in mice after *i.p.* injection [129]. In vivo studies by other workers also found that GO did not induce changes in the appearance of eyeball and intraocular pressure of rabbits [130], while amino-GO did not induce pulmonary thrombotoxicity in mice after intravenous injection [131].

On the contrary, pristine LPE-graphene was found to be toxic and distributed mainly in the brain and kidney of chicken embryos [132]. The finding was not surprising because LPE-graphene contained toxic solvent and surfactant residues, as mentioned above. Several in vivo studies in mice revealed that GO induces inflammatory response, pulmonary edema, and granuloma formation, especially at high concentrations. Moreover, dose-, size-, and time-dependent inflammation was found in the lung and liver, with cell infiltration and fibrosis [47,110,118,133,134,135]. For instance, Wang et al. reported that GOs with a low dose (0.1 mg) and medium dose (0.25 mg) had no obvious toxicity to Kunming (KM) mice after intravenous injection [47]. At a high dose of 0.4 mg per animal, chronic toxicity, such as mice death and lung granuloma formation were observed. The deaths occurred 1–7 days after intravenous injection. In addition, GOs were mainly located in the lung, liver and spleen. Zhang et al. indicated that the in vivo effect of GOs is dose-dependent. At a dose of 1 mg/kg, low uptake in the reticulo-epithelial system (RES) and no pathological changes were found in mice through intravenous injection for 14 days [134]. However, inflammation cell infiltration, pulmonary edema, and granuloma formation were observed in the lung at a dose of 10 mg/kg. Ma et al. administrated 5 mg/kg body weight of small GOs (50–350 nm) and large GOs (750–1300 nm) into balb/c male mice through *i.p.* and intratracheal instillation for three days [110]. They indicated that large GOs trigger increased the expression of inflammatory cytokines in the abdominal cavity and blood of mice after *i.p* injection. This led to enhanced recruitment of leukocytes in peritoneal cavities, resulting in acute inflammation. After intratracheal instillation, large GOs also caused a drastic increase in the production of pulmonary and systemic inflammatory cytokines, and the recruitment of inflammatory cells. More recently, Amrollahi-Sharifabadi et al. intraperitoneally injected GOs (5–10 μm) with doses of 50, 150, or 500 mg/kg into Wistar rats [135]. GOs first entered the blood and then localized in the liver. As known, bilirubin is a liver enzyme that can serve as a biomarker for liver damage. From hematological analysis, GOs with a dose of 500 mg/kg produced a significant increase in the serum level of bilirubin after 21 days, implying liver toxicity. Moreover, GOs induced inflammation and a granulomatous reaction in a dose-dependent manner. Xia and coworkers conducted oropharyngeal aspiration of GO, hGO, and rGO in C57BL/6 mice [87]. They reported that hydrated GO is more toxic than GO and rGO. hGO induced acute lung inflammation, accompanied by the highest lipid peroxidation in alveolar macrophages and cytokine production due to the formation of carbon radicals.

**Figure 19:**
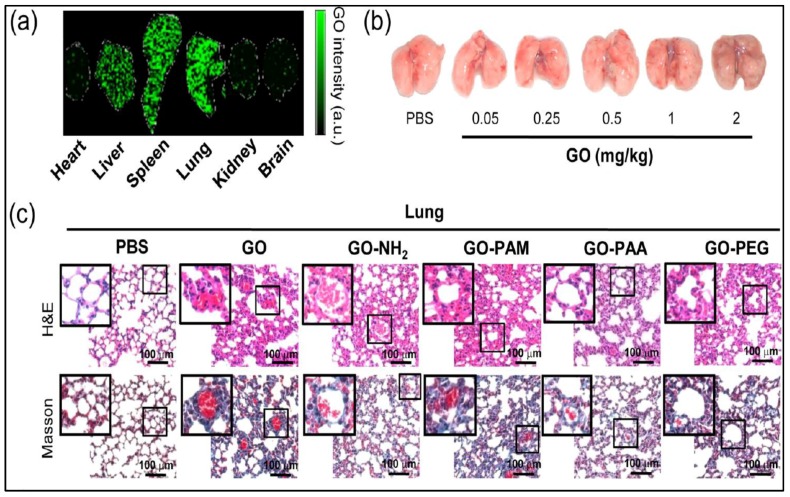

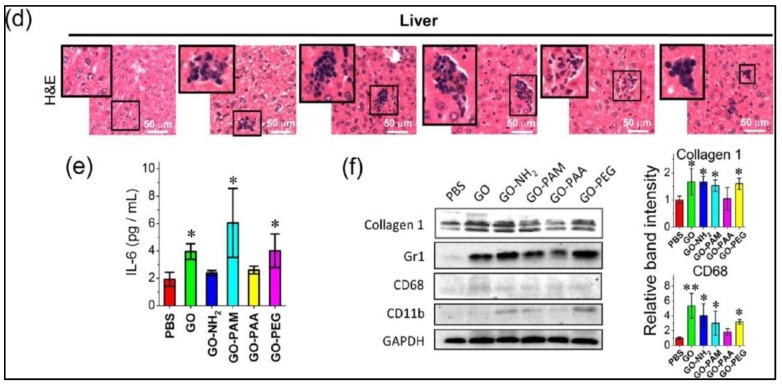
(**a**) Laser desorption/ionization mass spectrometry (LDIMS) image analysis showing biodistribution of GO in mice treated with GO (2 mg/kg body weight) for one day. (**b**) Images of mice lungs after treating with GO at 0.05, 0.25, 0.5, 1, and 2 mg/kg body weight for 1 day. Histological images of (**c**) lungs stained with H&E and Masson’s trichrome and (**d**) livers with H&E from mice injected with 1 mg/kg GO for 14 days. Magnified images from the line squares are the enlarged pulmonary alveoli. Dark spots are GO-cell complexes in livers. For Masson’s trichrome staining, blue color reveals collagen in lung. (**e**) IL-6 levels in sera from mice treated with a dose of 1 mg/kg body weight for 1 day (*n* = 5). (**f**) Protein markers of collagen 1, Gr1, CD68, and CD11b measured by Western blot analysis in lungs from mice treated at 1 mg/kg body weight for 14 days. Quantified data are depicted in the right panel (*n* = 4). * and ** imply *p* < 0.05 and *p* < 0.005 as compared to PBS-treated mice. Reproduced from [119] with permission of The American Chemical Society.

From in vitro cell cultivation, GO-PEG induced immune responses through cytokine secretion in murine macrophages. So, PEGylation did not help in passivating GO surfaces [117,118]. It is interesting to know whether GO-PEG sheets induce toxic effect in animal models in vivo. Recently, Xu et al. administered balb/c mice with GO, GO-PEG, GO-PAA, GO-NH_2_, and GO-PAM at 0.05, 1, 5, 10, and 20 mg/kg body weight through intratail vein injection [119]. The survival rate in mice was 100%, 80%, 60%, 20%, and 0%, respectively, at those prescribed GO doses. Mice administrated with GO at 1 mg/kg for 1 day and 14 days suffered from significant platelet depletion in peripheral blood. GO-PAM and GO-PEG also caused platelet depletion for one day, but no depletion was found in the GO-NH_2_ and GO-PAA. GOs were mainly accumulated in the lung, spleen, and liver, as indicated by laser desorption/ionization mass spectrometry (LDIMS) analysis (Figure 19a). The feature of lungs became darker with increasing GO dosages, implying enhanced agglomeration and accumulation of GOs (Figure 19b). Histological observations of lung and spleen organs were conducted using hematoxylin and eosin (H&E) and Masson’s trichrome staining (Figure 19c,d). In H&E staining, the nuclei of cells were stained blue by hematoxylin, whereas the cytoplasm and extracellular matrix were stained pink with eosin. GO and GO-PAM induced serious lung injuries, as evidenced by alveolar wall thickening due to infiltration by inflammatory cells, and increased collagen in bronchioles. However, GO-PAA caused lesser damage in lung and liver when compared to other mice treated with GO, GO-NH_2_, GO-PAM, and GO-PEG. Xu et al. attributed these differences to the distinct changes of IL-6 and collagen 1 contents because of fibrosis (Figure 19e,f and to the compositions of protein coronas. GO-PAA and GO-PEG contained lower immunoglobulin G content in their protein coronas (30−40%) than GO, GO-NH_2_, and GO-PAM (50−70%). From these, GO-PEG is moderately safer than GO in vivo, GO-PAA shows the best compatibility, and GO-PAM exhibits the highest cytotoxicty in vitro and in vivo.

Syama et al. administrated PrGO (rGO-PEG) with a dosage of 10 mg/kg body weight into Swiss Albino mice through intraperitoneal and intravenous injections for 3, 7, 14, and 21 days [136]. Confocal Raman microscopy was used to analyze the biodistribution of PrGO. Raman imaging of blood, urine, bone marrow, and other organs at the end of prescribed periods showed the presence of PrGO sheets in the brain, liver, kidney, spleen, and bone marrow. Urine Analysis revealed very weak PrGO intensity, demonstrating low renal excretion rate of PrGO through the kidney. The uptake of PrGO in the brain from both injection routes revealed that PrGO can cross the blood-brain barrier (BBB) and then accumulate in brain. Recently, Shang et al. administrated 4 mg/kg GOs into balb/c mice through *i.p.* injection. High levels of ROS and MDA due to lipid peroxidation were responsible for the brain and kidney damages [23]. Syama et al. also demonstrated that injection routes also affect the translocation of PrGO in mice organs [136]. PrGO injected intravenously translocated from the blood circulatory system into liver and spleen after three days. Maximum uptake of PrGO was found in liver and spleen, followed by kidney and brain. In the case of *i.p.* administration, PrGO was first adsorbed in the peritoneal cavity and then accumulated in liver. Repeated injection of PrGO caused acute liver injury and increased splenocyte proliferation. The splenocyte proliferation stimulated immune system responses due to the presence of a variety of cell types with different immune functions, such as macrophages, dendritic cells, B cells and T cells inside the spleen. Similarly, Mendonca et al. reported that rGO and rGO-PEG weaken the BBB junction of Wistar mice as a result of ROS and lipid peroxidation generation [117,137]. Increases in the antioxidant enzyme levels of catalase, SOD-1, and thiobarbituric acid reactive substances in hippocampus were responsible for oxidative stress generation. These findings indicated that surface functionalization of GOs and rGOs with PEG cannot fully eliminate cytotoxicity that is induced by graphene. More systematic studies of toxicity versus biocompatibility issues, especially in animal models, are needed to understand the biological effects and the safety of graphene nanomaterials to humans [138]. From the in vivo animal studies, ROS and oxidative stress appear to be the key factors in causing cell membrane damage upon exposure to GOs [23,136,137]. As is known, elevated ROS levels can disrupt lipids, proteins, and DNA, resulting in the membrane damage and eventual cell death [139]. Oxidative stress is therefore linked to a wide range of pathological anomalies and degenerative diseases, especially erythrocytes and brain cells that involve oxygen transport and aerobic respiration [140,141,142,143,144,145]. Table 2 presents a list of in vivo studies summarizing the different administration routes of GO-based nanomaterials and their associated biological effects.

## 4. GO-Polymer Nanocomposites

GOs can disperse well in water due to the presence of oxygenated groups. So, hydrolytic polymers, such as polyvinyl alcohol (PVA) and PEG, can react with GO readily in aqueous solution to form polymer nanocomposites [77]. Hydrogen bonding between the oxygenated groups of GO and PVA can prevent filler aggregation and promote polymer-filler interactions. When comparing with the pristine polymer, the mechanical strength of GO/PVA nanocomposites increases markedly due to the effective stress transfer mechanism from the polymer matrix to GO fillers during tensile testing [146]. Synthetic biodegradable polymers such as polylactic acid (PLA), polyglycolide (PGA), and poly(lactic-co-glycolic)acid (PLGA) copolymer find useful applications in biomedical field including drug delivery and bone tissue engineering as sutures and scaffolds [147,148]. PLA is a hydrophobic polymer due to the presence of −CH_3_ side groups in its repeated units. The methylene groups render PLA with a slow biodegradation rate, low mechanical strength, and toughness. Poor mechanical strength of PLA hinders its development for fabricating internal fixation devices for fracture bones in orthopedics. Biodegradable polymer fixation devices have a distinct advantage over their metallic counterparts because the devices can degrade in human body, so revision surgery to remove them is unnecessary. This greatly reduces the pain of patients suffering from bone fractures and the cost of hospital care. In recent years, the number of patients suffering from bone-related diseases, such as cancer and bone fracture, rises sharply due to an increase in older population and environmental pollution globally. In addition, sports and recreation activities also cause bone injuries in adults. There is an urgent need in biomedical sector to develop advanced biomaterials and polymer nanocomposites with good biocompatibility for bone tissue engineering applications. Over recent years, attention has focused on the improvement of biocompatibility and mechanical property of biodegradable polyesters that are reinforced with GOs [80,149]. The mechanical performance of PLA can be improved by adding low GO contents. This is because GO with higher elastic modulus (207.6 ± 23.4 GPa) is an effective reinforcement for PLA to form polymer nanocomposites. Furthermore, GO also promotes the adhesion and proliferation of bone cells (osteoblasts), as well as the differentiation of stem cells [150,151,152]. In this respect, GO/PLA biocomposites with good biocompatibility and tensile strength are ideal biomaterials for bone tissue engineering and regenerative medicine applications.

**Figure 20:**
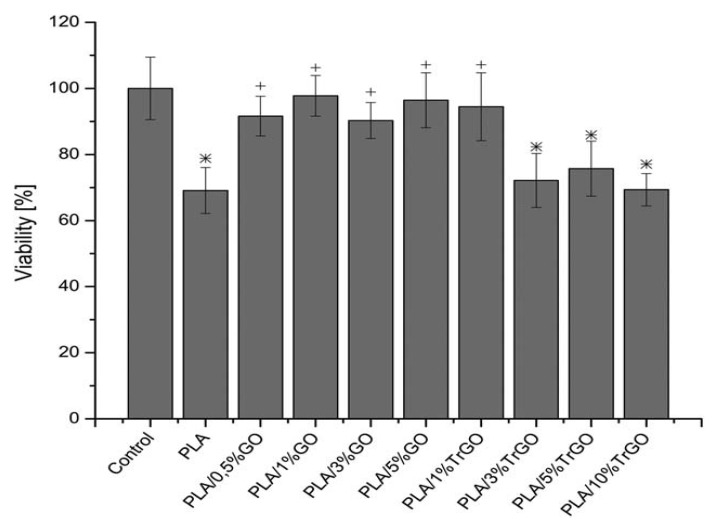
MTT results for PLA/GO and thermally reduced graphene oxide/polylactic acid (PLA/TRG) nanocomposites cultivated with Saos-2. * denotes statistically significance between experimental groups and control. + represents statistical significance between experimental groups and PLA (*n* = 6; *p* < 0.05). Reproduced from [81] with permission of Wiley.

Arriagada et al. studied the effects of GO (0.5–5 wt.%) and TRG additions (1–10 wt.%) on the biocompatibility of melt-mixed GO/PLA and PLA/TRG nanocomposites [81]. The human osteosarcoma cell line (Saos-2) was cultured on these nanocomposites. When comparing with pristine PLA, the addition of only 0.5 wt.% GO to PLA improves the viability of Saos-2 from less than 70% to about 90% (Figure 20). Cell viability can be further improved by adding 1 wt.% GO. This is because hydrophilic GO can enhance protein adsorption and subsequent cell adhesion. In the case of PLA/TRG nanocomposites, the viability of Saos-2 remains nearly the same with that of pure PLA, with the exception of PLA/1% TRG nanocomposite. Hydrophobic TRG does not enhance adhesion and proliferation of Saos-2. Considering mechanical properties, elastic modulus of PLA increases from about 2.1 GPa to 2.2 GPa and 2.4 GPa respectively by adding 1 wt.% and 2 wt.% GO, corresponding to 4.8% and 14.3% enhancement in the stiffness. Similarly, Pinto et al. also found that GO enhances the adhesion and proliferation of fibroblast on the surfaces of PLA/GO nanocomposite films [153]. It is noteworthy that suspensions of GOs with sharp edges can penetrate cell membrane and reach cytoplasm, leading to enhanced ROS level and eventual cell death, as mentioned previously. However, the polymeric matrix of polymer nanocomposites can seal sharp edges of GOs effectively, thereby preventing the damage of cell membrane from the GO penetration [15]. Because GOs are firmly bound to the polymeric matrix, so they cannot move freely to induce cell membrane damage and apoptosis. In this respect, GOs with large surface areas provide effective sites for the adhesion and proliferation of osteoblasts during cell cultivation.

Conventional hydrogels that were synthesized from using organic cross-linker have many drawbacks including poor absorption, mechanical and structural properties. Hydrogels are hydrophilic, three-dimensional networks of polymers that are capable of absorbing a large amount of water or biological fluids, thereby showing attractive applications in tissue engineering and drug delivery [154]. GO is known to be an effective reinforcement for hydrogel systems due to its abundant hydrophilic groups on the surface [155,156]. Thus GO can establish strong interfacial interactions with water soluble polymers (e.g., PVA, PEG and PAA) through chemical or hydrogen bonding [157,158,159,160,161,162]. For instance, Zhang et al. prepared GO/PVA composite hydrogel with improved tensile strength of 3.48 MPa by adding 0.8 wt% GO, being 132% increase than neat PVA hydrogel. Such nanocomposite hydrogel had a non-toxic effect on osteoblasts [157]. The introduction of PEG could lead to more efficient grafting of PVA molecules on the GO surface. As such, GO was exfoliated and uniformly dispersed in the PVA matrix of GO/PVA-PEG hydrogels [159]. Zhong et al. prepared GO/PAA nanocomposite hydrogels with an elongation at break of 2980% and tensile strength of 777 kPa, whose cross-linked network was facilitated by physical ion crosslinking (Fe^3+^ ions) [161]. Very recently, Jing et al. prepared self-healing rGO/PAA hydrogels having good biocompatibility with fibroblasts and high stretch ability, thus showing potential application for artificial skin devices [162].

## 5. Conclusions

In this review, we summarized and discussed the synthesis processes of graphene-based nanomaterials, their biocompatibility, and cytotoxicity. Furthermore, the in vitro and in vivo cytotoxicity of graphene-based nanomaterials were also addressed. The main challenge in graphene commercialization nowadays is to produce high quality graphene on a large scale and at low cost. The quality of graphene plays an important role in the biomedical field because the presence of impurities can have an adverse effect on mammalian cells by inducing cell membrane damage and apoptosis. Proper understanding of direct interactions between nanostructured graphene and mammalian cells can be obtained by using pure graphene. Although mechanical exfoliation of graphite flakes can produce pure graphene, the yield is very low for cell manipulation purposes. CVD-graphene is too expensive and its transferring process from copper or nickel foil to the target glass or SiO_2_/Si substrate is tedious. Nevertheless, high-quality CVD-graphene provides useful information relating its interactions with cells. Most in vitro cell culture studies revealed that CVD-graphene with a nanoscale dimension is biocompatible with several cell lines, especially enhancing fibroblast adhesion and promoting hMSCs differentiation into bone cells. These results are encouraging for potential applications of graphene in orthopedics. The preparation of LPE-graphene sheets requires toxic solvents and surfactants for graphite exfoliation. Those chemical reagents, especially the surfactants, are difficult to eliminate from final graphene sheets, thus the use of LPE-graphene in the biomedical field is limited. A typical example is the LPE-graphene induces cytotoxicity in the brain and kidney tissues of chicken embryos [132].

So far, GOs prepared by the modified Hummers process are widely used for investigating their interactions and responses with mammalian cells in vitro and in vivo. Conflicting results are found in the literature about the biocompatibility and cytoxicity of GOs and rGOs under in vitro and in vivo conditions. This is partly related to the researchers employing various oxidation times, different types, and different concentrations of oxidants for the synthesis, producing GOs with structure and reactivity as well as impurity level that differ from one study to another. The diversity in the structural properties has a large impact on the cell-GO interactions. In addition, the various chemical oxidizers and reducing agents used to prepare GOs and rGOS can generate metallic impurities and organic contaminations, thus altering their interactions with cells, tissues and organs, and resulting in cellular damage and apoptosis. In particular, toxic hydrazine reducing agent causes significant cell destructions in hMSCs at very low rGO content (1.0 μg/mL) [122], while residual Na_2_S in rGO induces serious pro-inflammatory responses in macrophages [133]. These adverse effects may partly explain the discrepancies in experimental results reporting in the literature. GOs and rGOs that were prepared from the green synthesis route are quite promising nanomaterials for biomedical applications, since they can reduce cytotoxicity to a low extent [105].

It is considered that the toxicity of GOs/rGOs can be minimized by dispersing them in polymers to form polymer nanocomposites. As such, graphene nanomaterials are immobilized since they are firmly bound to the polymer matrix of nanocomposites. In addition, the polymer matrix can seal the sharp edges of GOs/rGOs, preventing their penetration into cytoplasm. So, graphene nanofillers with large surface areas provide effective sites for cell adhesion and proliferation, especially for osteoblasts (bone cells). Furthermore, oxygenated groups of GOs can reduce the hydrophobicity of the polymeric matrix, thus facilitating cell attachment and spreading on the polymer surface as well [15,16]. This approach opens new opportunities for biomedical applications of graphene nanomaterials in orthopedics to make advanced bone fixation devices, scaffolds, and implants. Further safety evaluation and research must be carried out to ensure those polymer nanocomposites are biocompatible with human tissues prior to clinical applications.

## Figures and Tables

**Table 1:** In vitro cytotoxicity induced by graphene and its derivatives.

Material	Lateral Size, nm	Cell Type	Concentration and Exposure Time	Cytotoxic Effect	Ref.
CVD-graphene	Thickness: 3–5 layers	PC12	0.01, 0.1, 1, 10 and 100 µg/mL for 1–24 h	Apoptosis at ≥10 µg/mL	[89]
GO	200–700	RAW 264.7	1, 10, 50 and 200 µg/mL for 6 and 24 h	Apoptosis by membrane pores at ≥10 µg/mL	[84]
GO	200–700	A549	1, 10, 50 and 200 µg/mL for 6 and 24 h	Dose dependent toxicity. Cell death at ≥50 µg/mL	[84]
GO	979	Human Skin Keratinocyte	0.4, 1.2, 3.7, 11.1, 33.3 and 100 µg/mL for 3–72 h	Dose- and time-dependent ROS production	[88]
GO	342–765	Human Erythrocyte	3.125, 6.25, 12.5, 25, 50, 100 and 200 µg/mL for 3 h	Dose- and size-dependent hemolysis	[45]
GO	385	HepG2	1,2, 4, 8 and 16 µg/mL for 72 h	Plasma membrane damage at 4 µg/mL	[43]
GO	440–670	Swine Spermatozoa	0.5, 1, 5, 10 and 50 µg/mL for 1 to 4 h	Dose dependent toxicity. Cell death at ≥10 µg/mL	[82]
GO	---	hCorECs; hConECs	12.5, 25, 50 and 100 µg/mL for 2 h and 24 h	Apoptosis at ≥50 µg/mL	[113]
GO	201	Murine Macrophage	1, 2, 4, 10, 20, 50 and 100 µg/mL for 24 h	Cell membrane damage at ≥10 µg/mL	[119]
GO-PEG	10–120	Saos-2; MC3T3-E1; RAW-264.7	75 µg/mL for 24 h	GO-PEG accumulated on F-actin; ROS formation	[116]
GO-PEG	200	Murine Macrophage	10 and 40 µg/mL for 6, 12, 24 and 48 h	Inflammation response by secreting cytokine	[118]
GO-PAM	363	Murine Macrophage	1, 2, 4, 10, 20, 50 and 100 µg/mL for 24 h	Cell membrane damage at ≥10 µg/mL	[119]
rGO-PEG	910	Murine Astrocyte	10 and 100 µg/mL	Excess ROS and cell death at 100 µg/mL	[117]
rGO; rGO-PEG	---	A549	1–200 µg/mL	Dose dependent toxicity. Apoptosis at ≥25 µg/mL	[121]
Green rGO	65–90	Human Lymphocyte	50, 100 and 250 µg/mL	Loss of lysosomal integrity at ≥100 µg/mL	[105]
rGO	11	hMSCS	0.1 µg/mL	DNA fragmentation and chromosomal aberration	[122]
GO; rGO	400–800	HUVEC	10 µg/mL	GO induced more ROS, HO1 and TrxR levels, and DNA damage than rGO	[126]
hGO; rGO	105–150	BEAS-2B; THP-1	25, 50, 100 and 200 µg/mL for 24 h	hGO induced toxicity due to lipid peroxidation. rGO had little effect on cell viability	[87]
hGO; rGO	105–150	Murine Erythrocyte	25, 50, 100 and 200 µg/mL	rGO and hGO showed negligible and high rates of hemolysis respectively	[87]
TRG	---	Monkey Vero	10, 50, 100 and 300 µg/mL for 24 h	Apoptosis at >100 µg/mL	[127]

**Table 2:** In vivo studies showing different administration routes of GOs and functionalized GOs.

Material	Animal Model	Dosage	Administration Process	Biological Effect	Ref.
GO	Balb/c mice	4 mg/kg	Oral feeding and *i.p.* injection	Insignificant toxicity in mice	[128]
GO	Mice	50 µg/mouse	Intraperitoneal injection	No acute and chronic inflammation after intraperitoneal injection	[129]
GO	Rabbits	100–300 μg/eye	Intravitreal injection	No change in eyeball appearance and intraocular pressure	[130]
GO-PEG	Balb/c mice	20 mg/kg	Intravenous injection	low uptake by RES; no sign of toxicity on spleen and liver	[48]
GO-NH_2_	Mice	250 μg/kg	Intravenous injection	No pulmonary thromboembolism	[131]
GO	KM mice	0.1, 0.25 and 0.4 mg per mouse	Intravenous injection	GOs found in the lung, liver and spleen; Dose-dependent lung inflammation and granuloma	[47]
hGO; GO	B6 mice	2 mg/kg	Oropharyngeal aspiration	Hydrated GOs induced more serious lung inflammation & lipid peroxidation in alveolar macrophages than GOs	[87]
GO	Balb/c mice	4 mg/kg	Intraperitoneal injection	GOs induced brain and kidney damages by increasing ROS and MDA, but decreasing glutathione levels	[23]
GO	Balb/c mice	5 mg/kg	Intravenous & intratracheal administration	Large GOs (750–1300 nm) induced very high pulmonary and systemic inflammatory cytokine production and inflammatory cell recruitment	[110]
GO	KM mice	10 mg/kg	Intratracheal instillation	GOs mainly retained in the lung. Acute lung injury and chronic pulmonary fibrosis.	[133]
GO	Mice	10 mg/kg	Intravenous injection	Inflammation cell infiltration, pulmonary edema and granuloma formation in the lung	[134]
GO	Wistar rat	50, 150, or 500 mg/kg	Intraperitoneal injection	Granulomatous reaction with giant cell formation; neuronal degeneration and necrosis	[135]
rGO	Wistar rat	7 mg/kg	Intravenous injection	rGO entered hippocampus & thalamus, reduced paracellular tightness of BBB	[137]
rGO-PEG	Wistar rat	7 mg/kg	Intravenous injection	rGO-PEG reduced blood-brain barrier function due to ROS and lipid peroxidation generation	[117]
rGO-PEG	Albino mice	10 mg/kg	Intravenous and *i.p.* injections	rGO-PEG distributes in liver, kidney bone marrow, spleen and brain	[136]
